# Downregulation of circ-ZNF609 Promotes Heart Repair by Modulating RNA N^6^-Methyladenosine-Modified *Yap* Expression

**DOI:** 10.34133/2022/9825916

**Published:** 2022-04-07

**Authors:** Lijun Wang, Pujiao Yu, Jiaqi Wang, Guie Xu, Tianhui Wang, Jingyi Feng, Yihua Bei, Jiahong Xu, Hongbao Wang, Saumya Das, Junjie Xiao

**Affiliations:** ^1^Cardiac Regeneration and Ageing Lab, Institute of Geriatrics (Shanghai University), Affiliated Nantong Hospital of Shanghai University (The Sixth People's Hospital of Nantong), School of Medicine, Shanghai University, Nantong 226011, China; ^2^Shanghai Engineering Research Center of Organ Repair, School of Life Science, Shanghai University, Shanghai 200444, China; ^3^Department of Cardiology, Tongji Hospital, Tongji University School of Medicine, Shanghai 200065, China; ^4^Department of Cardiology, Yangpu Hospital, Tongji University School of Medicine, Shanghai 200065, China; ^5^Cardiovascular Division of the Massachusetts General Hospital and Harvard Medical School, Boston, MA 02114, USA

## Abstract

Circular RNAs take crucial roles in several pathophysiological processes. The regulatory role and its underlying mechanisms of circ-ZNF609 in the heart remains largely unknown. Here, we report that circ-ZNF609 is upregulated during myocardial ischemia/reperfusion (I/R) remodeling. Knockdown of circ-ZNF609 protects against acute I/R injury and attenuates left ventricle dysfunction after I/R remodeling *in vivo*. *In vitro*, circ-ZNF609 regulates cardiomyocyte survival and proliferation via modulating the crosstalk between Hippo-YAP and Akt signaling. Mechanically, N^6^-methyladenosine-modification is involved in the regulatory role of circ-ZNF609 on YAP. An in-depth study indicates that knockdown of circ-ZNF609 decreases the expression of YTHDF3 and further fine-tuned the accessibility of *Yap* mRNA to YTHDF1 and YTHDF2 to regulate YAP expression. circ-ZNF609 knockdown represents a promising therapeutic strategy to combat the pathological process of myocardial I/R injury.

## 1. Introduction

Cardiovascular disease is one of the leading causes of death worldwide, with data suggesting increasing morbidity every year due to a higher incidence of heart failure. Myocardial infarction (MI) caused by coronary thrombosis is the source of tremendous socioeconomic burden in most countries [1–3]. Although modern reperfusion treatment is able to repair the injured heart and reduce the mortality of MI patients at the acute phase, many of these survivors will still suffer from adverse remodeling and the downstream consequences of heart failure [4–6]. In the past couple of decades, many molecules and signaling pathways have been identified as potential therapeutic targets to promote beneficial remodeling in post-MI patients [7–9]. Classical clinical drugs, such as *β* receptor blocker, angiotensin-converting enzyme (ACE) inhibitors, and aldosterone receptor antagonists, were demonstrated to reverse cardiac fibrosis and ameliorate remodeling in post-MI patients [10]. However, therapies targeting cardiac repair to improve outcomes for those patients are still an unmet need.

Circular RNA (circRNA) is a class of newly identified endogenous RNAs that are generated by forming 3′,5′-phosphodiester bonds across the junction site [11]. circRNAs are widely distributed in human tissues and are important regulators in several diseases and pathophysiological processes, including cancer, neurological diseases, and cardiovascular diseases [12, 13]. RNA N^6^-methyladenosine (m^6^A) methylation is the most abundant modification of RNAs, and aberrant m^6^A methylation is closely related to many diseases [14]. In cardiovascular diseases, several circRNAs have been identified and reported to participate in essential processes of cardiovascular development and diseases [15–21]. Although m^6^A modifications on circRNAs have been identified to be closely related to diseases, such as cancer and innate immunity, the role of circRNAs with m^6^A modification in the heart remains largely unknown [22–26]. circ-ZNF609, also known as myocardial infarction-associated circular RNA (MICRA), was identified with RNA m^6^A modification and found to have functions in myoblast, rhabdomyosarcoma, and vascular endothelium [27–30]. The MICRA level in MI patients' peripheral blood is reported to be associated with outcomes after MI clinically [31, 32]. However, the role of circ-ZNF609 in heart remains largely unknown.

Here, we explored the regulatory role of circ-ZNF609 in myocardial ischemia/reperfusion (I/R) injury by using murine together with neonatal rat cardiomyocyte (NRCM) models. *In vitro*, circ-ZNF609 regulates cardiomyocyte survival via modulating the crosstalk between Hippo-YAP and Akt signaling. Mechanistically, circ-ZNF609 regulates YAP through interaction with YTHDF3 in cardiomyocytes. Knockdown of circ-ZNF609 leads to the decrease of YTHDF3 and, therefore, tunes the accessibility of *Yap* mRNA to YTHDF1 and YTHDF2, consequently regulating the expression of YAP. Our study provides new insights into our mechanistic understanding of circRNA function and suggests that inhibition of circ-ZNF609 promotes heart repair after I/R injury by modulating RNA m^6^A-modified *Yap* expression. circ-ZNF609 knockdown may be a promising therapeutic strategy to combat the pathological process of myocardial I/R injury.

## 2. Results

### 2.1. circ-ZNF609 Is Upregulated during Myocardial I/R Injury

**Figure 1:**
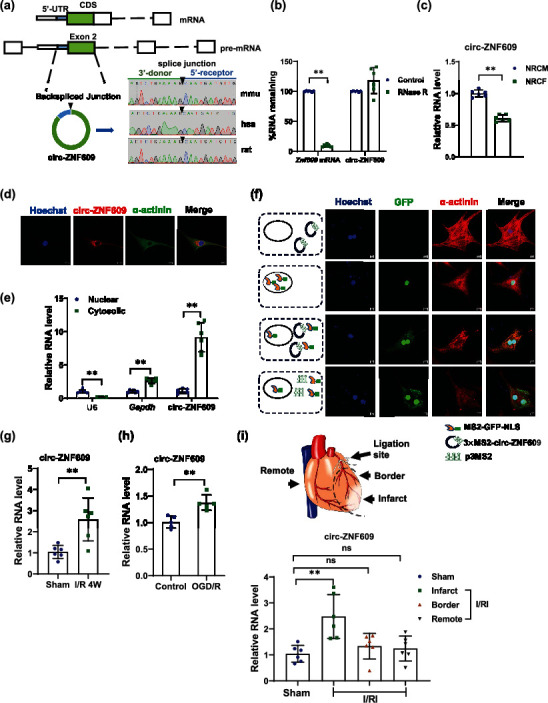
circ-ZNF609 is upregulated during myocardial I/R injury. (a) The expression of circ-ZNF609 was validated by RT-PCR followed by Sanger sequencing. mmu: total RNA extracted from mouse heart; rat: total RNA extracted from rat heart; hsa: total RNA extracted from AC16 cardiomyocyte cell line. (b) qRT-PCR for the abundance of circ-ZNF609 and *Zfp609* mRNA in mouse hearts treated with RNase R (^∗∗^*P* < 0.01. *n* = 6 wells/group). (c) Expression of circ-ZNF609 in NRCMs (neonatal rat cardiomyocytes) compared to NRCFs (neonatal rat cardiac fibroblasts) (^∗∗^*P* < 0.01, *n* = 6 wells/group). (d) RNA fluorescence in situ hybridization assay (FISH) of circ-ZNF609 in NRCM (scale bar = 10 *μ*m). (e) qRT-PCR indicates the abundance of circ-ZNF609 in the cytoplasm or nucleus (^∗∗^*P* < 0.01, *n* = 6 wells/group). (f) circ-ZNF609 localized in the cytoplasm of cardiomyocytes, as evidenced by MS2-tagged circ-ZNF609 cotransfection with MS2-GFP-NLS (scale bar = 10 *μ*m). (g) Increased circ-ZNF609 in murine hearts from 4 weeks post-I/R injury versus sham control (^∗∗^*P* < 0.01, *n* = 6 mice/group). (h) The expression of circ-ZNF609 was increased in OGD/R-induced NRCM apoptosis model (^∗∗^*P* < 0.01 wells, *n* = 5/group). (i) Top: the schematic diagram of definition of cardiac infarct, border, and remote zones. Bottom: the expression level of circ-ZNF609 was analyzed by qRT-PCR in the infarct, border, and remote zones of mouse acute I/R hearts compared to the sham group (^∗∗^*P* < 0.01, ns: nonstatistically significant. *n* = 6 mice/group). NRCM: neonatal rat cardiomyocyte; I/R: ischemia/reperfusion; OGD/R: oxygen-glucose deprivation/reperfusion. Data are presented as means ± S.D. (b, c, e, g–i) Independent-sample *t*-test.

To explore the potential role of circ-ZNF609 in the heart, we firstly use divergent PCR primers, which specifically amplify circ-ZNF609 following Sanger sequencing to verify the existence of circ-ZNF609 (Figure 1(a)). Then, we assessed the stability of circ-ZNF609, as shown in Figure 1(b) and Supplementary Figure S1a; circ-ZNF609 is more resistant to RNase R exonuclease treatment and actinomycin D treatment than mRNA. The expression of circ-ZNF609 was widely expressed in adult mice, and the abundance of circ-ZNF609 was found at an intermediate level in the heart (Supplementary Figure S1b). Then, we examined the conservation of circ-ZNF609 across mammalian species and found that circ-ZNF609 was highly conserved in mice, rats, and humans (Supplementary Figure S2). qRT-PCR results showed that primary neonatal rat cardiomyocyte (NRCM) expressed a higher level of circ-ZNF609 compared to neonatal rat cardiac fibroblasts (NRCFs) (Figure 1(c)). Moreover, qRT-PCR and RNA fluorescence in situ hybridization assay demonstrated that circ-ZNF609 prefers to locate in the cytoplasm (Figures 1(d) and 1(e)). We next designed an MS2-tagged circ-ZNF609 to validate the localization of circ-ZNF609 using a modified MS2-GFP system, as shown in Figure 1(f); in this system, MS2-GFP-NLS localizes in the nucleus of cardiomyocytes, but cotransfection of MS2-GFP-NLS with p3MS2 plasmids, which express cytoplasmic localized 3×MS2 RNA and could bind the MS2-GFP-NLS, led to the distribution of GFP in the cytoplasm. After the cotransfection of the MS2-tagged circ-ZNF609 plasmids, we found that GFP distributed into the cytoplasm of cardiomyocytes, confirming that circ-ZNF609 localized in the cytoplasm of cardiomyocytes. Collectively, these data suggest that circ-ZNF609 is a conserved, stable, and abundantly expressed cytoplasmic circular RNA in cardiomyocytes.

Next, we evaluated the expression of circ-ZNF609 by qRT-PCR in an established murine model of cardiac I/R injury. After cardiac ischemia and 4 weeks of remodeling (chronic ischemic HF), circ-ZNF609 was significantly increased in the heart tissue from surgery-operated mice in contrast to the sham control (Figure 1(g)). A similar change of circ-ZNF609 occurred in the NRCMs with oxygen-glucose deprivation/reperfusion (OGD/R) treatment, a cellular model of an oxidative stress injury to mimic I/R injury *in vitro* (Figure 1(h)). Also, circ-ZNF609 was significantly increased in the infarct zone of acute I/R (30 min/24 hrs) (Figure 1(i)). Taken together, these data suggest a possible involvement of circ-ZNF609 in modulating myocardial I/R injury.

### 2.2. Knockdown of circ-ZNF609 Protects against Acute Myocardial I/R *In Vivo*

**Figure 2:**
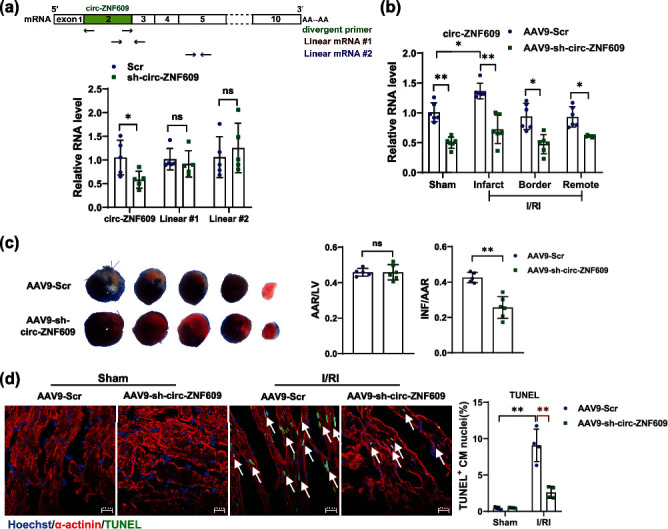
Knockdown of circ-ZNF609 protects against acute myocardial I/R *in vivo*. (a) sh-circ-ZNF609 specifically knock down circ-ZNF609 without affecting the expression of linear *Zfp609* (human ortholog named as *Znf609*) mRNA (^∗^*P* < 0.05. ns: nonstatistically significant. *n* = 5 wells/group). (b) Expression of circ-ZNF609 in different zones of the heart from mice treated with AAV9-sh-circ-ZNF609 (^∗^*P* < 0.05,  ^∗∗^*P* < 0.01, *n* = 6 mice/group). (c) Attenuated infarct size in heart treated with AAV9-sh-circ-ZNF609 after ischemia/reperfusion injury (I/RI), as evidenced by 2,3,5-triphenyltetrazolium chloride (TTC) staining (^∗∗^*P* < 0.01; ns: nonstatistically significant. *n* = 5 and 6 mice, respectively). (d) Decreased myocardial apoptosis in the mouse I/RI heart treated with AAV9-sh-circ-ZNF609, as evidenced by TUNEL staining (^∗∗^*P* < 0.01, *n* = 4 mice/group, scale bar = 20 *μ*m). I/R: ischemia/reperfusion; TUNEL: terminal deoxynucleotidyl transferase dUTP nick end labeling. Data are presented as means ± S.D. (a, c) Independent-sample *t*-test; (b, d) two-way ANOVA test.

To determine the biological function of circ-ZNF609 during myocardial I/R injury, we generated an shRNA of circ-ZNF609 (sh-circ-ZNF609) which specifically ablates circ-ZNF609 without affecting the level of linear *Znf609* (*Zfp609* in mouse) mRNA (Figure 2(a)). We then administrated AAV9-sh-circ-ZNF609 or its scrambled control (AAV9-Scr) in mice through tail-vein injection to suppress circ-ZNF609 *in vivo*. One week after AAV9 injection, coronary artery ligation for 30 min followed by 24 hr reperfusion was performed to induce cardiac I/R injury in mice. qRT-PCR analysis for circ-ZNF609 confirmed that circ-ZNF609 was downregulated in the heart after AAV9-sh-circ-ZNF609 administration (Figure 2(b)). By performing TTC (2,3,5-triphenyltetrazolium chloride) staining and TUNEL (terminal deoxynucleotidyl transferase dUTP nick end labeling) staining, significant reduction in infarct size and decrease in cardiomyocyte apoptosis were observed in the acute I/R mice treated with AAV9-sh-circ-ZNF609 compared with scrambled control (Figures 2(c) and 2(d)). These results suggest that inhibition of circ-ZNF609 can prevent against cardiac I/R injury *in vivo*.

### 2.3. Knockdown of circ-ZNF609 Attenuates LV Dysfunction after I/R Remodeling *In Vivo*

**Figure 3:**
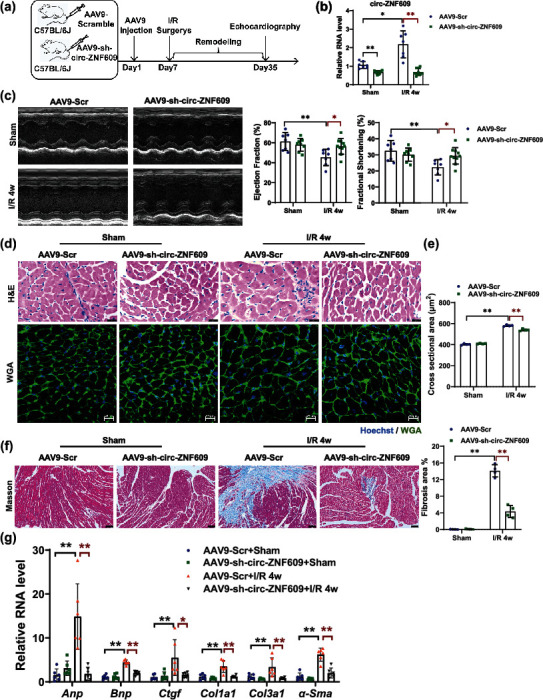
Knockdown of circ-ZNF609 attenuates LV dysfunction after I/R remodeling *in vivo*. (a) The schedule of virus injection and myocardial I/R injury-induced mouse pathological cardiac remodeling model establishment. (b) qRT-PCR showed circ-ZNF609 knockdown in mouse hearts via tail-vein injection with AAV9-sh-circ-ZNF609 (^∗^*P* < 0.05,  ^∗∗^*P* < 0.01, *n* = 7, 8, 7, and 10 mice, respectively). (c) Preserved left ventricular ejection fraction (EF) and fractional shortening (FS) in I/R remodeling for 4 weeks of hearts from mice treated with AAV9-sh-circ-ZNF609, as evidenced by echocardiography (^∗^*P* < 0.05,  ^∗∗^*P* < 0.01, *n* = 7, 8, 7, and 10 mice, respectively). (d) Representative images of H&E staining (top, scale bar = 25 *μ*m) and WGA staining (bottom, scale bar = 20 *μ*m). (e) Quantitative analysis of cross-sectional area of cardiac cells stained with WGA at indicated groups (^∗∗^*P* < 0.01, *n* = 4 mice/group). (f) Reduced cardiac fibrosis in I/R remodeling for 4 weeks of hearts from mice treated with AAV9-sh-circ-ZNF609, as evidenced by Masson's staining (^∗∗^*P* < 0.01, *n* = 4 mice/group, scale bar = 75 *μ*m). (g) Decreased expression of both the pathological hypertrophic genes (*Anp*, *Bnp*) and fibrotic genes (*α-Sma*, *Ctgf*, *Col1a1*, and *Col3a1*) in I/R remodeling for 4 weeks of hearts from mice treated with AAV9-sh-circ-ZNF609 (^∗^*P* < 0.05,  ^∗∗^*P* < 0.01, *n* = 6 mice/group). LV: left ventricular; I/R: ischemia/reperfusion; H&E: hematoxylin-eosin; WGA: wheat germ agglutinin. Data are presented as means ± S.D. (b, c, e–g) Two-way ANOVA test.

The cardiac protective effect which was exhibited by circ-ZNF609 knockdown after acute I/R injury prompted us to examine whether this beneficial effect could exist in the pathological process of cardiac remodeling after I/R injury. After AAV9-sh-circ-ZNF609 or AAV9-scrambled control (AAV9-Scr) were transfused via tail-vein injection, I/R or sham surgery was conducted on mouse hearts 7 days later (Figure 3(a)). A significant decrease of circ-ZNF609 in hearts was demonstrated in the murine hearts treated with AAV9-sh-circ-ZNF609 (Figure 3(b)). As measured by echocardiography, knockdown of circ-ZNF609 preserved heart function as indicated by preserved ejection fraction (EF) and fractional shortening (FS) at 4 weeks after I/R surgery (Figure 3(c)). Hematoxylin and eosin (H&E) and wheat germ agglutinin (WGA) staining revealed that the hypertrophied ventricular myocardial cells and larger interstitial between cardiomyocytes during I/R remodeling were prevented by AAV9-sh-circ-ZNF609 administration (Figures 3(d) and 3(e)). Meanwhile, cardiac fibrosis was also attenuated by circ-ZNF609 knockdown in the I/R group assessed by Masson's Trichrome staining (Figure 3(f)). Consistently, both the pathological hypertrophic genes (*Anp*, *Bnp*) and fibrotic genes (*α-Sma*, *Ctgf*, *Col1a1*, and *Col3a1*) were decreased after AAV9-sh-circ-ZNF609 administration (Figure 3(g)). Taken together, knockdown of circ-ZNF609 is able to protect against myocardial I/R remodeling via improving cardiac function and reducing cardiac fibrosis.

### 2.4. Downregulation of circ-ZNF609 Contributes to Cardiomyocyte Survival *In Vitro*

**Figure 4:**
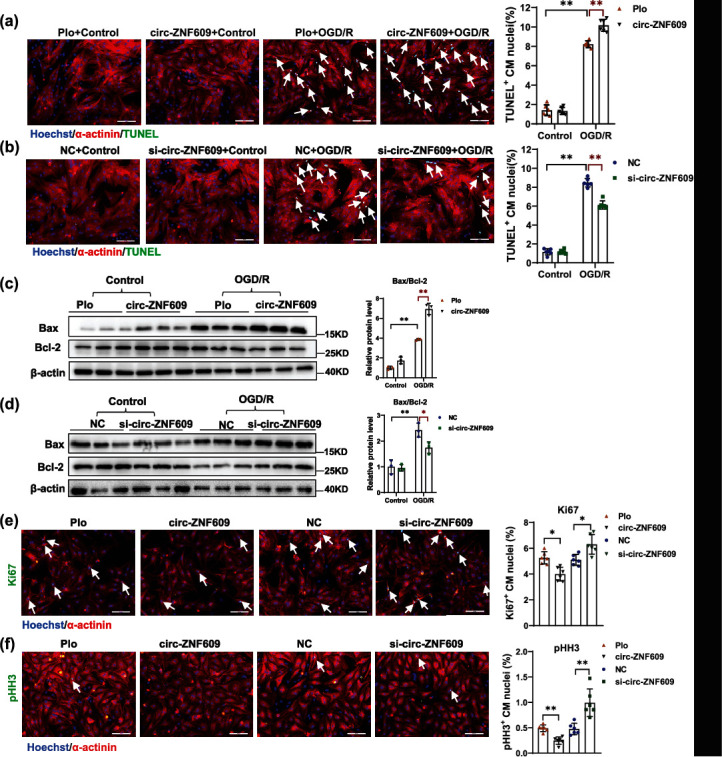
Downregulation of circ-ZNF609 contributes to cardiomyocyte survival *in vitro*. (a, b) Representative images of immunofluorescence staining and quantification of the relative TUNEL-positive NRCMs showed that circ-ZNF609 regulates NRCM apoptosis (^∗∗^*P* < 0.01, *n* = 6 wells/group, scale bar = 100 *μ*m). (c, d) Western blot analysis of NRCM apoptosis by detection of Bax and Bcl-2 in OGD/R-induced apoptosis model treated with or without circ-ZNF609 overexpression or knockdown (^∗^*P* < 0.05,  ^∗∗^*P* < 0.01, *n* = 3 wells/group). (e, f) Representative images of immunofluorescence staining and quantification of the relative Ki67-positive and pHH3-positive cardiomyocytes treated with or without circ-ZNF609 overexpression or knockdown (^∗^*P* < 0.05,  ^∗∗^*P* < 0.01, *n* = 6 wells/group, scale bar = 100 *μ*m). NC: siRNA negative control; si-circ-ZNF609: siRNA targeted to circ-ZNF609; Plo: circRNA overexpression empty vector Plo-ciR without insert circ-ZNF609 sequence; circ-ZNF609: circ-ZNF609 overexpression construct; NRCM: neonatal rat cardiomyocyte; OGD/R: oxygen-glucose deprivation/reperfusion. Data are presented as means ± S.D. (a–d) Two-way ANOVA test; (e, f) independent-sample *t*-test.

Next, we explored the effect of circ-ZNF609 on primary NRCM in culture. We generated a circ-ZNF609 overexpression construct that was transiently transfected into NRCMs; the overexpression efficiency and precise cyclization in the back-splicing junction were verified by qRT-PCR, agarose gel, and Sanger sequencing (Supplementary Figure S3). The siRNA which was able to specifically knockdown circ-ZNF609 was used as previously reported [29]. Overexpression of circ-ZNF609 aggravated NRCM apoptosis while knockdown of circ-ZNF609 led to a significant reduction of cardiomyocyte apoptosis as revealed by TUNEL staining (Figures 4(a) and 4(b)) and western blots (Bax/Bcl-2) (Figures 4(c) and 4(d)). As previously reported, promoting cardiomyocyte proliferation is along with significant restoration after myocardial injury in adult hearts [33, 34]. We therefore explored the influence of circ-ZNF609 in the regulation of markers of cell cycle on cardiomyocytes. As demonstrated in Figures 4(e) and 4(f), immunostaining for histone H3 phosphorylated at serine 10 (pHH3) and Ki67 showed that silencing circ-ZNF609 increased the percentage of pHH3-positive or Ki67-positive NRCMs compared to control, while overexpression of circ-ZNF609 significantly inhibited proportion of NRCMs with pHH3 and Ki67 positive. Thus, these data indicate that the knockdown of circ-ZNF609 promotes cardiomyocyte survival.

### 2.5. circ-ZNF609 Regulates Cardiomyocyte Survival through Modulating the Crosstalk between Hippo-YAP and Akt Signaling

**Figure 5:**
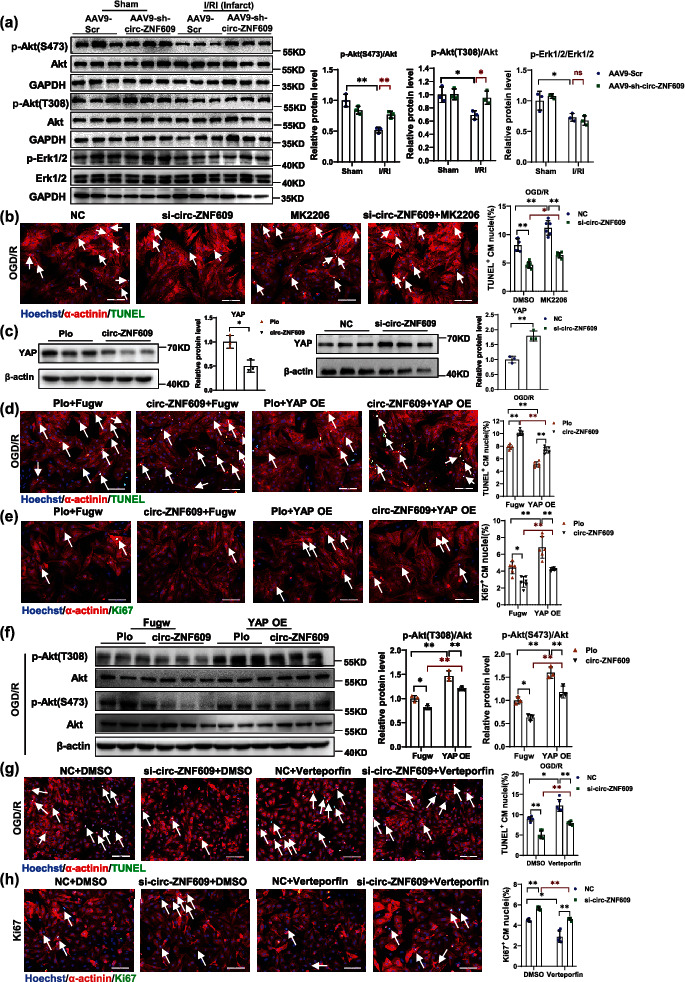
circ-ZNF609 regulates cardiomyocyte survival via modulating the crosstalk between Hippo-YAP and Akt signaling. (a) Western blot analysis demonstrated the Akt and Erk phosphorylation levels in acute I/R heart treated with AAV9-sh-circ-ZNF609 or scrambled control (^∗^*P* < 0.05,  ^∗∗^*P* < 0.01; ns: nonstatistically significant; *n* = 3 mice/group). (b) Representative images of immunofluorescence staining and quantification of the relative TUNEL-positive NRCMs showed that treatment with the Akt inhibitor MK2206 partially blunt the protective effect of circ-ZNF609 knockdown (^∗^*P* < 0.05,  ^∗∗^*P* < 0.01, *n* = 6 wells/group. Scale bar = 100 *μ*m). (c) Western blot analysis of total YAP in cardiomyocytes after transfection with si-circ-ZNF609 or circ-ZNF609 (^∗^*P* < 0.05,  ^∗∗^*P* < 0.01, *n* = 3 wells/group). (d) Representative images of immunofluorescence staining and quantification of the relative TUNEL-positive NRCMs showed that YAP overexpression could block the proapoptosis effect of circ-ZNF609 (^∗∗^*P* < 0.01, *n* = 6 wells/group, scale bar = 100 *μ*m). (e) Representative images of immunofluorescence staining and quantification of the relative Ki67-positive NRCMs showed that YAP overexpression could significantly blunt the decrease of the proportion of cardiomyocytes with Ki67 after circ-ZNF609 overexpression (^∗^*P* < 0.05,  ^∗∗^*P* < 0.01, *n* = 6 wells/group, scale bar = 100 *μ*m). (f) Forced expression of YAP would blunt the inhibition of Akt signaling after circ-ZNF609 overexpression in the OGD/R-induced NRCM apoptosis model (^∗^*P* < 0.05,  ^∗∗^*P* < 0.01, *n* = 3 wells/group). (g, h) Representative images of immunofluorescence staining and quantification of the relative TUNEL-positive (g) and Ki67-positive (h) NRCMs showed that YAP/TEAD inhibitor verteporfin could blunt the prosurvival effects of circ-ZNF609 knockdown in NRCM (^∗^*P* < 0.05,  ^∗∗^*P* < 0.01, *n* = 6 wells/group, scale bar = 100 *μ*m). NC: siRNA negative control; Plo: circRNA overexpression empty vector Plo-ciR without insert circ-ZNF609 sequence; circ-ZNF609: circ-ZNF609 overexpression construct; Fugw: empty vector without YAP overexpression; YAP OE: YAP overexpression construct; OGD/R: oxygen-glucose deprivation/reperfusion. Data are presented as means ± S.D. (a, b, d–h) Two-way ANOVA test; (c) independent-sample *t*-test.

Akt activation and Erk1/2 activation have been considered to take a cardioprotective role in response to I/R injury [35, 36]. Thus, we asked whether the regulatory role of circ-ZNF609 is involved in regulating Akt and Erk1/2. We detected the level of phospho-Akt in acute I/R injury cardiac tissues. As evidenced by western blot, the phosphorylation level of Akt (both in Ser 473 and Thr 308) and Erk1/2 was significantly downregulated in the acute I/R injury heart, while knockdown of circ-ZNF609 dramatically elevated the Akt (Ser 473 and Thr 308) but not Erk1/2 phosphorylation level (Figure 5(a)). Similar observations regarding the effect of circ-ZNF609 ablation on Akt activation but not Erk1/2 were also noted in the *in vitro* OGD/R-induced NRCM apoptosis model (Supplementary Figure S4). Meanwhile, TUNEL staining showed that Akt inhibitor MK2206 can partially blunt the protective effect of knockdown circ-ZNF609 against OGD/R-induced NRCM apoptosis (Figure 5(b)). These data suggest that Akt signaling is implicated in the regulatory role of circ-ZNF609 in cardiomyocytes.

The interesting phenomena that circ-ZNF609 regulates Akt activation but not Erk1/2 in cardiomyocytes promoted us to seek for the specific mediator across Akt signaling and circ-ZNF609. The Hippo effector, transcriptional coactivator Yes-associated protein (YAP), expression causes an increase in Akt activation without a change in Erk activation in cardiomyocytes [37]. YAP has been found to activate PI3K-Akt through targeting Pik3cb with TEAD [37]. Therefore, we tested the hypothesis that YAP might be the mediator between circ-ZNF609 and Akt signaling. As evidenced by western blots in Figure 5(c) and Supplementary Figure S5a, circ-ZNF609 could negatively regulate endogenous YAP expression and positively regulate the phosphorylation level (S127) of YAP in NRCM. Also, these negative regulatory effects of circ-ZNF609 in Hippo-YAP signaling were also observed in AAV9-sh-circ-ZNF609-treated mouse hearts after I/R remodeling (Supplementary Figure S5b). Moreover, overexpression of YAP could alleviate OGD/R-induced NRCM apoptosis (Supplementary Figures S5c-S5d). Hippo effector YAP overexpression would blunt the antisurvival effects of circ-ZNF609 overexpression in cardiomyocytes as evidenced by TUNEL staining and immunostaining for Ki67 (Figures 5(d) and 5(e)). Importantly, forced expression of YAP would blunt the inhibition of Akt signaling after circ-ZNF609 overexpression in OGD/R cardiomyocytes (Figure 5(f)). It was previously reported that YAP/TEAD is the mechanistic link between YAP and AKT signaling [37]. We then detected the expression of TEAD1 in circ-ZNF609 knockdown heart tissues, as determined by western blot in Supplementary Figure S5e; silencing circ-ZNF609 did not affect the protein level of TEAD1. YAP nuclear translocation is important for its transcriptional activation; thus, we examined whether circ-ZNF609 affect the cellular localization of YAP. Immunofluorescence staining and western blot of YAP on the cytoplasmic and nuclear fractions revealed that increased nuclear accumulation of YAP was observed after circ-ZNF609 knockdown, suggesting that circ-ZNF609 can regulate the nuclear translocation of YAP in NRCM (Supplementary Figures S5f-S5g). We further used two kinds of YAP/TEAD inhibitors, small molecule compound inhibitor verteporfin and peptide inhibitor peptide 17, to block the interaction between YAP and TEAD. As shown in Figures 5(g) and 5(h) and Supplementary Figure S6, verteporfin and peptide 17 could both blunt the prosurvival effects of circ-ZNF609 knockdown in NRCM. Collectively, these data suggested that the crosstalk between Hippo-YAP and Akt signaling is modulated by circ-ZNF609 to regulate cardiomyocyte survival.

### 2.6. circ-ZNF609 Regulates YAP Expression via YTHDF3 in Cardiomyocytes

**Figure 6:**
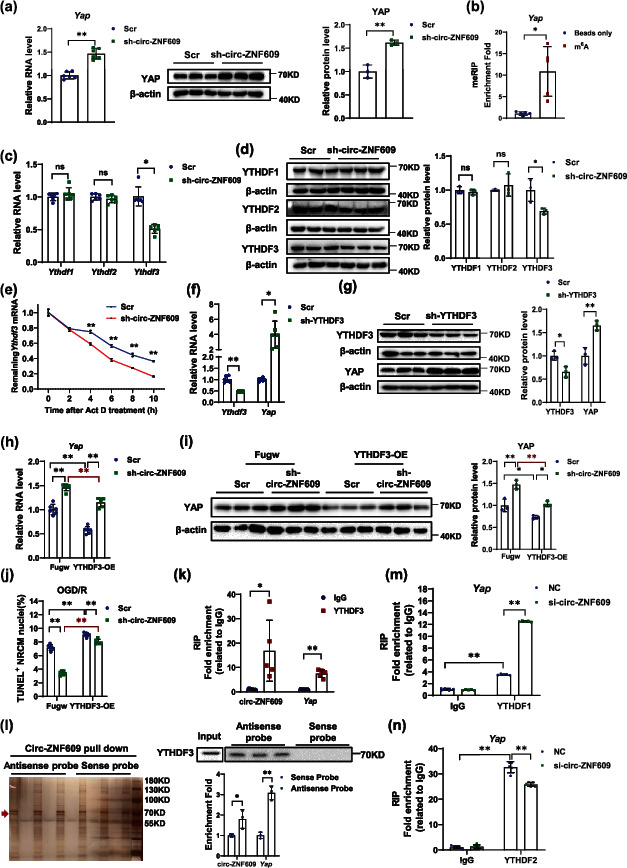
circ-ZNF609 regulates YAP expression via YTHDF3 in cardiomyocytes. (a) The expression of circ-ZNF609 was validated by qRT-PCR (^∗∗^*P* < 0.01, *n* = 6 wells/group) and western blot (^∗∗^*P* < 0.01, *n* = 3 wells/group). (b) Validation of m^6^A modification in *Yap* mRNA using meRIP-qPCR (^∗^*P* < 0.05, *n* = 5/group). (c) qRT-PCR demonstrated the expression level of *Ythdf1*, *Ythdf2*, and *Ythdf3* treated with sh-circ-ZNF609 (^∗^*P* < 0.05; ns: nonstatistically significant. *n* = 6 wells/group). (d) Western blot analysis demonstrated the YTHDF1, YTHDF2, and YTHDF3 expression levels treated with sh-circ-ZNF609 (^∗^*P* < 0.05; ns: nonstatistically significant. *n* = 3 wells/group). (e) The amount of *Ythdf3* mRNA in AC16 cardiomyocytes treated with actinomycin D with or without circ-ZNF609 knockdown at indicated time points (^∗∗^*P* < 0.01 at each time point, *n* = 6/group). (f) qRT-PCR demonstrated the expression level of *Yap* with or without YTHDF3 knockdown (^∗^*P* < 0.05,  ^∗∗^*P* < 0.01, *n* = 6 wells/group). (g) Western blot analysis demonstrated the expression level of YAP with or without YTHDF3 knockdown (^∗^*P* < 0.05,  ^∗∗^*P* < 0.01, *n* = 3 wells/group). (h) qRT-PCR demonstrated the expression levels of *Yap* at indicated groups (^∗∗^*P* < 0.01, n = 6 wells/group). (i) Western blot demonstrated the expression of levels of YAP at indicated groups (^∗^*P* < 0.05,  ^∗∗^*P* < 0.01, *n* = 3 wells/group). (j) Quantification of the relative TUNEL-positive NRCMs showed that YTHDF3 overexpression could block the protective effect of circ-ZNF609 knockdown (^∗∗^*P* < 0.01.*n* = 6 wells/group). (k) RNA immunoprecipitation assay demonstrated YTHDF3 binding to circ-ZNF609 and *Yap* (^∗^*P* < 0.05,  ^∗∗^*P* < 0.01, *n* = 5/group). (l) Left: silver staining of the circ-ZNF609-protein complex pulled down by circ-ZNF609 junction antisense probe or sense probe with cell lysates from AC16 cardiomyocytes. Right: western blot and qRT-PCR analysis suggested that YTHDF3 protein and *Yap* mRNA were specifically pulled down by circ-ZNF609 antisense probe (^∗^*P* < 0.05,  ^∗∗^*P* < 0.01, *n* = 3/group). (m) RNA immunoprecipitation assay demonstrated the binding activity of YTHDF1 to *Yap* mRNA with or without circ-ZNF609 knockdown (^∗∗^*P* < 0.01, *n* = 5/group). (n) RNA immunoprecipitation assay demonstrated the binding activity of YTHDF2 to *Yap* mRNA with or without circ-ZNF609 knockdown (^∗∗^*P* < 0.01, *n* = 5/group). Scr: shRNA scrambled control; sh-circ-ZNF609: shRNA targeted to circ-ZNF609; Fugw: empty vector without YTHDF3 overexpression; sh-YTHDF3: shRNA targeted to YTHDF3; YTHDF3: YTHDF3 overexpression construct; NC: siRNA negative control; si-circ-ZNF609: siRNA targeted to circ-ZNF609; OGD/R: oxygen-glucose deprivation/reperfusion. (a–g, k, l) Independent-sample *t*-test; (h–j, m, n) two-way ANOVA test.

Next, we asked how circ-ZNF609 regulate YAP expression in cardiomyocytes. circ-ZNF609 has been reported to have N^6^-methyladenosine (m^6^A) modification [27]. Using the RMVar database, potential m^6^A modification motifs were identified across coding sequences of *Yap* mRNA [38]. Therefore, we asked whether RNA m^6^A modification contributes to the regulation effects of circ-ZNF609 on YAP. The negative regulation of circ-ZNF609 on YAP was confirmed in the AC16 cardiomyocyte cell line (Figure 6(a)). Next, m^6^A methylated RNA immunoprecipitation (meRIP) was conducted to confirm the occurrence of m^6^A in *Yap* mRNA (Figure 6(b)). The m^6^A modification, which was recognized by YTHDFs, has been reported to mediate two different mRNA fates by altering the mRNA decay or mRNA translation [39]. Thus, we investigated whether the YTHDF proteins were involved in the function of circ-ZNF609. As shown in Figures 6(c) and 6(d), YTHDF3 was downregulated both at the RNA and protein levels upon circ-ZNF609 knockdown, while the expression level of YTHDF1 and YTHDF2 was not regulated by circ-ZNF609 alteration. Further, the actinomycin D treatment assay revealed that silencing circ-ZNF609 could promote the mRNA degradation of *Ythdf3* (Figure 6(e)). We therefore propose that YTHDF3 is dominantly involved in the regulation role of circ-ZNF609 in cardiomyocytes. Consistently, YTHDF3 negatively regulated the expression of YAP (Figures 6(f) and 6(g)). Also, the negative regulatory effect of circ-ZNF609 on YAP was blunted via YTHDF3 (Figures 6(h) and 6(i)). Further, overexpression of YTHDF3 in NRCM abolished the protective effects of circ-ZNF609 suppression in OGD/R-induced cardiomyocyte apoptosis (Figure 6(j)). It is previously reported that YTHDF3 could facilitate translation and degradation of m^6^A-modified RNA and modulate the accessibility of mRNA to YTHDF1 and YTHDF2 [39]. YTHDF1 and YTHDF2 function in promoting translation and RNA degradation [40, 41]. Considering that circ-ZNF609 regulated the expression of YAP through YTHDF3, we raised our hypothesis that downregulated YTHDF3 expression affected the binding activity of *Yap* mRNA to YTHDF1 and YTHDF2; YTHDF3 function as a binding partner of YTHDF1 to facilitate YAP translation or competitively with YTHDF2 to access *Yap* mRNA. We thus performed RNA immunoprecipitation (RIP) assays and revealed the binding of circ-ZNF609 and YAP mRNA to YTHDF3 protein (Figure 6(k)). The binding of circ-ZNF609, *Yap* mRNA, and YTHDF3 was further verified by RNA pull down; *Yap* mRNA and YTHDF3 protein were specifically pulled down by circ-ZNF609 junction antisense probe compared to sense probe (Figure 6(l)). Then, we explored whether YTHDF proteins function as the cooperative partner to YTHDF3 in regulating YAP. Interestingly, the binding of *Yap* mRNA to YTHDF1 was significantly increased after circ-ZNF609 knockdown, while the binding of *Yap* mRNA to YTHDF2 was decreased (Figures 6(m) and 6(n)). These data indicate that circ-ZNF609 altered the expression of YAP through YTHDF3 in cardiomyocytes. Mechanically, YTHDF3 modulated the accessibility of *Yap* mRNA to YTHDF1 and YTHDF2 and, consequently, regulate the expression of YAP.

## 3. Discussion

**Figure 7:**
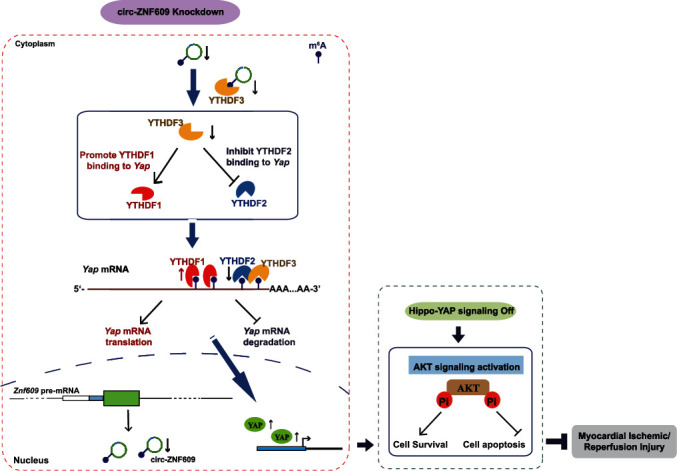
Schematic for our proposed mechanism of circ-ZNF609 in regulating heart repair. Downregulation of circ-ZNF609 contributes to heart repair via modulating the crosstalk between Hippo-YAP and Akt signaling. Mechanistically, circ-ZNF609 binds to YTHDF3, and RNA m^6^A modification is involved in the regulatory role of circ-ZNF609 in YAP. Knockdown of circ-ZNF609 decreases the expression of YTHDF3 and further fine-tuned the accessibility of *Yap* mRNA to YTHDF1 and YTHDF2 to regulate YAP expression.

Myocardial I/R injury and scar formation can occur in the MI patients who received reperfusion therapy leading eventually to ischemic HF and myocardial fibrosis. It is the main cause of recent major adverse cardiovascular events and results in long-term adverse prognosis in post-MI patients [4, 10]. Myocardial I/R injury is triggered by a sequence of progression pathological processes [3, 42–45]. However, effective strategies to attenuate myocardial I/R injury are still not available. In this study, we demonstrate that circ-ZNF609 is upregulated in I/R remodeling hearts and OGD/R treated cardiomyocytes. Knockdown of circ-ZNF609 in mouse hearts demonstrates cardioprotective effects on both acute I/R injury and I/R remodeling for 4 weeks. Downregulation of circ-ZNF609 contributes to cardiomyocytes' survival in NRCMs. circ-ZNF609 regulates cardiomyocyte survival via modulating the crosstalk between Hippo-YAP and Akt signaling. Inhibition of YAP/TEAD interaction (verteporfin or peptide 17), which disrupts the crosstalk between Hippo-YAP and Akt, would abolish the prosurvival effects of circ-ZNF609 knockdown. Mechanistically, circ-ZNF609 binds to YTHDF3, and RNA m^6^A modification is involved in the regulatory role of circ-ZNF609 in YAP nuclear translocation. Knockdown of circ-ZNF609 decreases the expression of YTHDF3 and further fine-tuned the accessibility of *Yap* mRNA to YTHDF1 and YTHDF2 to regulate YAP expression (Figure 7). Our study reveals the role of circ-ZNF609 in myocardial I/R injury; circ-ZNF609 knockdown represents a promising therapeutic target to combat the pathological process of myocardial I/R injury.

Efforts on RNA-based therapeutic strategies have garnered great interest lately [46]. Studies on circular RNAs have been discovered and involved in heart diseases [17–19, 21, 47–49]. However, the comprehensive function and its underlying mechanism in the heart remain largely unexplored. Here, we reported that circ-ZNF609 was upregulated in mouse myocardial I/R injury, and downregulation of circ-ZNF609 in the heart could promote heart repair by modulating the crosstalk between Hippo-YAP and Akt signaling. In peripheral blood, circ-ZNF609 was associated with LV dysfunction in MI patients and might be acting as a biomarker clinically [32]. Interestingly, the trend of circ-ZNF609 changes in the heart tissue is not correlated with that in patients' blood as previous clinical studies reported [31, 32]. This inconsistency between body fluids and tissues has also been reported in other diseases [50, 51]. The level of RNA in the circulation system is complexly regulated, and circ-ZNF609 in peripheral blood might be due to the extracellular vesicle transport between cells and tissues [52]. It is also possible that the accumulation of circ-ZNF609 in the heart tissues and reduction of circ-ZNF609 secretion led to this opposite observation. circ-ZNF609 was found to have functions in myoblast, rhabdomyosarcoma, and vascular endothelium via acting as miRNA sponges or capable of translation [28–30]. Our findings here uncover a critical role of circ-ZNF609 in myocardial I/R injury; it was found that in cardiomyocytes, circ-ZNF609 took a critical role in modulating the crosstalk between Hippo-YAP and Akt signaling. It was reported that circ-ZNF609 can be translated into a protein [29]. To examine whether the observed effects of circ-ZNF609 were from circRNA itself or from the circ-ZNF609 coded protein, we generated circ-ZNF609 mutant (rat-circ-ZNF609*Δ*1-2 and its human ortholog hs-circ-ZNF609*Δ*1-2) with deleted ATGs as previously reported, which lost the ability to encode proteins to evaluate the regulatory function of circ-ZNF609 [29]. The immunostaining of Ki67 and TUNEL staining indicated that the effects of wild-type rat-circ-ZNF609 on regulating NRCM proliferation and OGD/R-induced apoptosis still existed after rat-circ-ZNF609*Δ*1-2 mutant construct transfection (Supplementary Figures S7a-S7b). Meanwhile, its regulatory effects on YAP were also preserved in hs-circ-ZNF609*Δ*1-2 mutant compared to wild-type hs-circ-ZNF609 as revealed by qRT-PCR and western blot in AC16 cardiomyocytes (Supplementary Figures S7c-S7d). Thus, these results suggest that the observed effects of circ-ZNF609 on cardiomyocytes in this study most probably came from circRNA itself instead of the circ-ZNF609 coded protein. However, we cannot exclude the possibility that circ-ZNF609 encoded protein might exert its regulatory effects on cardiomyocytes in other stimulus conditions. The role of circ-ZNF609 coded protein in the hearts is yet to be determined and required further investigations. Collectively, these data provided an insightful role of circ-ZNF609 in the heart, especially in myocardial I/R injury and in OGD/R cardiomyocytes.

The Hippo-YAP pathway was first identified in Drosophila by genetic screens as a regulator of cell proliferation and organ size [8]. The core mammalian Hippo signaling components include the series kinases MST1/2, LATS1/2, and the scaffolding proteins MOB1. The Hippo downstream effector YAP has been proved to interact with transcription factors and stimulate cell proliferation in many organs including the heart [53–55]. The phosphoinositide 3-kinase- (PI3K-) Akt pathway is one of the most classical regulation pathways in cardiomyocytes [9, 56]. PI3K-Akt pathway activation protects against myocardial I/R injury and delays the progression of post-MI cardiac remodeling [36]. Recent studies indicated that YAP could activate PI3K-Akt by targeting Pik3cb with TEAD to promote cardiomyocyte survival [37]. In our study, we found that circ-ZNF609 modulated the crosstalk between Hippo-YAP and Akt signaling through the Hippo effector YAP. Forced expression of YAP would blunt the activation of Akt signaling after circ-ZNF609 knockdown in oxygen-glucose deprivation/reperfusion cardiomyocytes. As a signal integrator, the Hippo-YAP signaling pathway takes vital functions in cardiac homeostasis maintenance [57]. A very recent study demonstrated that direct chronic downregulation of Hippo in WW45cKO mice promotes pressure overload-induced cardiac dysfunction [58]. Those observations suggest that Hippo-YAP signaling has both detrimental and salutary functions in heart [58]. In this study, we reported that circ-ZNF609 modulated the activity of Hippo-YAP signaling in response to cardiomyocyte injury, indicating that circ-ZNF609 might be a potential therapeutic target for cardiovascular diseases. Currently, functional study on circ-ZNF609 *in vivo* is conducted by regulating the expression of circ-ZNF609 before ischemia injury happens. Further studies should be significant to transform these intervention methods of preconditioning into an actual therapeutic strategy to meet the demands of clinical application. In this study, a further in-depth study suggested that circ-ZNF609 regulated the expression of YAP through directly interacting with RNA m^6^A “reader” protein YTHDF3. RNA m^6^A methylation was involved in the regulatory role of circ-ZNF609 on YAP. The expression of YTHDF3 has been proposed to act as a “buffering agent” for target access to YTHDF1 and YTHDF2 [39]. Using RIP assays, we found that silencing circ-ZNF609 would affect the binding activity of *Yap* mRNA to YTHDF1 and YTHDF2. Downregulation of YTHDF3 by circ-ZNF609 suppression facilitated the binding activity of YTHDF1 and inhibited the access of YTHDF2 to *Yap* mRNA, therefore further regulating the expression level of YAP. RNA m^6^A is a conservative posttranscriptional modification; addressing the recognition specificity and regulation specificity of m^6^A modification has attracted lots of attention. In our present study, we uncovered the crucial regulatory role of circ-ZNF609 in cardiomyocytes, which might act as a key “bridge” for RNA m^6^A modification recognition and regulation. For one thing, circ-ZNF609 itself could be methylated by m^6^A modification and directly bind to the “reader” protein YTHDF3. For another, circ-ZNF609 could regulate the expression of YTHDF3, therefore modulating the accessibility of YTHDFs to m^6^A-modified mRNA *Yap*. Those observations suggested that circ-ZNF609 regulated the expression of YAP through balancing the recognition of YTHDF proteins to *Yap* mRNA. To our knowledge, this is the first report about m^6^A-modified circRNA exerting its regulatory role via regulating another m^6^A-modified mRNA expression, demonstrating the critical role of RNA m^6^A modification in circRNA regulatory function.

Currently, the most widely used methods to identify the cellular localization of circRNA are either isolating nuclear RNA and cytoplasmic RNA which is detected by qRT-PCR or RNA fluorescence in situ hybridization which is visualized by microscopy. However, due to the very high sequence similarity between circRNA and its derived linear mRNA, the base-pair probe-based RNA fluorescence in situ hybridization method applied to circRNA has some limitations. The probe used across the back-spliced site of circRNA is the only unique sequence region. Considering the length of the probe and the relative abundance of circRNA to its linear mRNA, RNA FISH applied to circRNA should be carefully designed with sufficient control samples. The MS2-MCP system has been widely used to visualize mRNA localization [59, 60]. However, this imaging system has not been applied in circRNAs. Here, in this study, we use a modified MS2-MCP system to visualize circ-ZNF609 cellular localization. MS2-GFP-NLS was localized in the nucleus because of the NLS signaling sequence. This modified MS2-MCP system validated the localization of circRNA based on the very specific interaction between MCP and MS2 aptamer-tagged transcript. MS2-GFP-NLS was exported to cytoplasm due to binding to 3×MS2-tagged circ-ZNF609; thus, GFP would be visualized in the cytoplasm, demonstrating that circ-ZNF609 is localized in the cytoplasm of cardiomyocytes. Different from the integrated MS2 sequences into the genome to imaging mRNA, in this study, the modified MS2-GFP system that was used to visualize the cellular localization of circRNA relied on exogenous expression of 3×MS2-tagged circ-ZNF609. Besides, this imaging method is limited to visualizing circRNAs in the cytoplasm, as the NLS is added to restrict MS2-GFP protein in the nucleus. Future efforts should be made to optimize this imaging system and apply this approach to image nuclear circRNAs. Though with the above limitation, this method could be an additional approach to cross-validate the cellular localization of circRNA regardless of specific probe design.

In conclusion, our study shows that knockdown of circ-ZNF609 contributes to heart repair via modulating the crosstalk between Hippo-YAP and Akt signaling. These data uncover a critical role of circ-ZNF609 in myocardial I/R injury, and circ-ZNF609 is important to modulate the binding activity of YTHDF proteins to *Yap* mRNA via interacting with YTHDF3 in an RNA m^6^A modification-dependent manner in cardiomyocytes.

## 4. Materials and Methods

### 4.1. Animals

All experiments of animals were in accordance with the guidelines on the use and care of laboratory animals for biomedical research published by the National Institutes of Health (No. 85-23, revised 1996) and approved by the committee on the Ethics of Animal Experiments of Shanghai University. Male C57BL/6J mice aged 7-8 weeks were purchased from Charles River Laboratories (Beijing, China) and raised in a specific pathogen-free (SPF) laboratory animal facility of Shanghai University (Shanghai, China).

### 4.2. Cardiac Ischemia/Reperfusion (I/R) Injury Mouse Model

To study the role of circ-ZNF609 in I/R injury, mice were randomized into two different groups, which were injected with AAV9-sh-circ-ZNF609 (10^13^ vg/mL, 30 *μ*L) or AAV9-scrambled control (10^13^ vg/mL, 30 *μ*L) via the tail vein a week before surgical operation. To establish the mouse cardiac I/R injury model, male mice aged 8 weeks with body weight of 23-26 g were anesthetized with intraperitoneal injection of 4% chloral hydrate. The anesthetized mice were intubated with a 24 G peripheral intravenous catheter after tracheotomy while a respirator was used to mimic artificial respiration and a homeothermic blanket was used to maintain body temperature at 37°C. Then, the left thoracic cavity was opened with microscissors, and the left anterior descending coronary artery (LAD) was stitched with 7-0 silk sutures to induce ischemia. After 30 min of myocardial ischemia, the stitch was removed to create reperfusion. The chest was closed with 5-0 silk sutures, and the tracheotomy intubation was extubated. The sham group underwent the same surgical procedures without LAD stitching. For acute I/R injury, the mouse heart was divided into the risk zone (anteroapical wall distal to the left anterior descending ligation site representing the infarct zone and left anterior wall close to the ligation site representing border zone) and remote zone (the site without ligation). All surgeries and analyses were performed by investigators blinded to the treatment.

### 4.3. Echocardiography

After 4 weeks of remodeling, mice were anesthetized by inhalation of 1.5-2% isoflurane. Cardiac function parameters of each mouse were detected by Vevo 2100 echocardiography (VisualSonics Inc, Toronto, Ontario, Canada) with a 30 MHz central frequency scan head. M-mode images were taken from the parasternal short-axis view at the level of papillary muscles. Left ventricular ejection fraction (LVEF) and left ventricular fractional shortening (LVFS) were measured. All the echocardiography data have been presented in Table S1. The echocardiographer was blinded to the surgical procedure and group.

### 4.4. Neonatal Rat Cardiomyocyte Isolation, Culture, and Treatment

Neonatal rat cardiomyocytes were isolated from the ventricular myocardium of 1- to 3-day-old SD rats by enzymatic digestion as previously described [35] and cultured in DMEM (Corning, USA) with 10% horse serum (Gibco, Grand Island, USA) and 5% fetal bovine serum (FBS, BioInd, Israel). All assays and transfections on NRCM were conducted in DMEM with 1% FBS. Plasmids (1 *μ*g/mL) and siRNA (100 nM) were transfected with either the Sinofection Transfection Reagent (Sino Biological, China) or Lipofectamine 2000 (Invitrogen, USA) for 72 h. NRCMs were treated with Akt inhibitor MK2206 (10 *μ*M, 24 h, Selleck), YAP-TEAD inhibitor verteporfin (250 nM, 24 h, Selleck), and YAP-TEAD inhibitor peptide 17 (50 nM, 24 h, Selleck) as indicated in the appropriate assays, respectively. To create OGD/R injury, cardiomyocytes were cultured in serum-free no glucose DMEM with a humidified hypoxic atmosphere containing 95% N_2_ and 5% CO_2_ at 37°C for 8 h followed by replacing the normal culture DMEM medium containing serum and glucose and then transferring to a normal incubator for 12 hr recovery.

### 4.5. Immunochemistry and Immunofluorescence Staining

Sections (5 *μ*m) and NRCMs were fixed, permeabilized, and blocked. Then, the sections or NRCMs were incubated with primary antibodies: *α*-actinin antibody (1 : 200, Sigma), Ki67 antibody (1 : 200, Abcam), and phospho-histone H3 (Ser10) polyclonal antibody (1 : 100, Invitrogen). After incubation of secondary antibodies, Hoechst (1 : 1000, Keygen) was used to label the nucleus for 30 min. The images of NRCMs were collected with a fluorescence microscope (Leica, Wetzlar, Germany), and the images of heart sample sections were collected by a confocal microscope (Carl Zeiss, Thuringia, Germany). All analyses were performed by investigators blinded to the treatment.

### 4.6. TUNEL Staining

To perform TUNEL staining, a DeadEnd Fluorometric TUNEL System (Promega Corp, Madison, USA, REF G3250) was used for frozen heart sample sections while an apoptosis detection kit (Vazyme, Nanjing, China, A111-03) was used for NRCMs. The staining was performed according to the instruction of the manufacturer's protocol. Cardiomyocytes were labeled with *α*-actinin while the nucleus was labeled with Hoechst as mentioned in the method of immunofluorescence staining. The images of NRCMs were collected with a fluorescence microscope (Leica, Wetzlar, Germany), and the images of heart sample sections were captured by a confocal microscope (Carl Zeiss, Thuringia, Germany).

### 4.7. TTC Staining

After 24 hours' reperfusion, LAD was religated at the same location, followed by injection of Evan's Blue (Sigma Aldrich, Saint Louis, USA) until the dye was visible on the skin of mice. Then, the heart sample was excised and sliced into 5-7 slices. The slices were incubated in 1% 2,3,5-triphenyltetrazolium chloride (Sigma Aldrich, Germany) and then transferred into 4% paraformaldehyde (PFA). Images of each slice were taken by a digital camera. The infarct area (INF, stained white) and area-at-risk (AAR, stained red in nonblue area) were measured by ImageJ software (National Institutes of Health).

### 4.8. Masson's Trichrome Staining and H&E Staining

To assess fibrosis and hypertrophy of heart tissue in mice, 5 *μ*m thick tissue sections of the heart sample were analyzed by Masson's Trichrome staining kit (Keygen Biotech, China) and H&E staining (Keygen Biotech, China). Images of each section were taken by the Nikon model (200x magnification) with the Spot Insight camera. Fibrosis areas (blue color) and whole section areas were analyzed by ImageJ software (National Institutes of Health), and the percentage of fibrosis was measured as fibrosis areas/whole section areas × 100%.

### 4.9. Wheat Germ Agglutinin (WGA) Staining

5 *μ*m frozen heart sections were fixed and stained with wheat germ agglutinin Alexa Fluor 488 Conjugate (Invitrogen). Images were taken by a confocal microscope (Carl Zeiss, Thuringia, Germany). ImageJ software was used to quantify cell size.

### 4.10. Quantitative Real-Time Polymerase Chain Reactions (qRT-PCRs)

Total RNA was extracted from tissue samples or cells using the TRIzol reagent (Invitrogen). The RNA was converted to cDNA using the PrimeScript RT reagent Kit with gDNA Eraser (RR047B, Takara, Japan) or TransScript-gDNA Removal and cDNA Synthesis SuperMix (AT311, Transgen, China). For the RNA stability assay, AC16 cardiomyocyte cells were seeded in six-well plates and incubated with actinomycin D (Selleck) for the time course. Total RNA was isolated, and then, the degradation rate of mRNA was analyzed by quantitative real-time PCR. Quantitative real-time PCR amplification was conducted by using SYBR Green (Takara, Japan) in the LightCycler 480 Real-Time PCR System (Roche, Basel, Switzerland). 18 s was used as an internal control for gene expressions. Primary sequences used in this study are listed in Table S2.

### 4.11. Western Blot

Protein concentrations of cells and tissue lysed by RIPA buffer (Beyotime Biotechnology, Nantong, China) were evaluated by the Takara BCA Protein Assay Kit. Equal amounts of protein samples were subjected to SDS-PAGE and transferred to polyvinylidene difluoride membranes. After being blocked by 5% nonfat dry milk in a Tris-buffered saline with Tween 20 buffer, membranes were incubated with the primary antibodies from the following source: Bax (1 : 1000, Abclonal, A12009), Bcl-2 (1 : 1000, Abclonal, A11025), *β*-actin (1 : 1000, Huabio, M1210-2), p-Akt (Ser 473) (1 : 1000, Cell signal technology, #4060), p-Akt (Thr 308) (1 : 1000, Cell signal technology, #13038), total-Akt (1 : 1000, Cell signal technology, #9272), YAP (1 : 1000, Cell signal technology, #14074), YTHDF1 (1 : 1000, Proteintech; 17479-1-AP), YTHDF2 (1 : 1000, Proteintech; 24744-1-AP), YTHDF3 (1 : 1000, Proteintech; 25537-1-AP, and 1 : 1000, Abcam; ab220161), Erk1/2 (1 : 1000, Cell signal technology, #4695), p-Erk1/2 (1 : 1000, Cell signal technology, #4370), MST1 (1 : 1000, Cell signal technology, #3682), MST2 (1 : 1000, Cell signal technology, #3952), WW45 (1 : 1000, Cell signal technology, #13301), LATS1 (1 : 1000, Cell signal technology, #3477), histone H3 (1 : 1000, Beyotime, AF0009), p-YAP (Ser127) (1 : 1000, Cell signal technology, #13008), and TEAD1 (1 : 1000, Abclonal, A6768). To perform nucleocytoplasmic separation, a Nuclear and Cytoplasmic Protein Extraction Kit (Keygen Biotech, #KGP1100) was used for NRCMs according to the instruction of the manufacturer's protocol. All proteins were visualized by the ECL Chemiluminescence kit (Tanon, China) and visualized using the Tanon-5200S Chemiluminescent Imaging System (Tanon, China). Band intensity was calculated by ImageJ.

### 4.12. Vector Construction

To generate the circ-ZNF609 overexpression vector, the cDNA sequence of circ-ZNF609 was amplified from rat cDNA using TransStart FastPfu Fly DNA Polymerase (AP231-12, TransGen). The PCR products were inserted into commercial circRNA overexpression plasmid Plo-ciR (CS0103, Geneseed). To generate circ-ZNF609 mutant, the pEASY-Uni Seamless Cloning and Assembly Kit (TransGen Biotech, #CU101) was used to obtain rat-circ-ZNF609*Δ*1-2 and its human ortholog hs-circ-ZNF609*Δ*1-2. For rat-circ-ZNF609*Δ*1-2, the primers used to generate PCR fragments used for recombination were the following: r-circ-ZNF609-d1d2-F: 5′-AGGTGGACGTTGACTCTAAGTCCTTGAGCAGTGGAGCCTG-3′ and r-circ-ZNF609-d1d2-R: 5′-CCCACCTCCTTGGAGCCTGAttCCAGTTTCTGCTGGTCCTTTT-3′ and r-circ-ZNF609-plo-op-R: 5′-CTTAGAGTCAACGTCCACCTCAGGAT-3′ and r-circ-ZNF609-plo-op-F: 5′-TCAGGCTCCAAGGAGGTGGGGA-3′. For hs-circ-ZNF609*Δ*1-2, the primers used to generate PCR fragments used for recombination were the following: hs-circ-ZNF609-d1d2-F: 5′-AGGTGGGACGTTGACTCTAAGTCCTTGAGCAGTGGAGCCT-3′ and hs-circ-ZNF609-d1d2-R: 5′-CCCACCTCCTTTGAGCCTGAttCCAGTTTCTGCTGGTCCTTT-3′ and hs-circ-ZNF609-plo-op-R: 5′-CTTAGAGTCAACGTCCCACCTCAAGA-3′ and hs-circ-ZNF609-plo-op-F: 5′-TCAGGCTCAAAGGAGGTGGGGATA-3′. To generate p3MS2 plasmids, 3×MS2 DNA sequence 5′-CGGGATCCGATATCCGTACACCATCAGGGTACGAGCTAGCCCATGGCGTACACCATCAGGGTACGACTAGTAGATCTCGTACACCATCAGGGTACGGAATTCTCTAGAGC-3′ was inserted into the pcDNA3.0 vector. To generate 3×MS2-circ-ZNF609, 3×MS2 DNA sequence was inserted into circ-ZNF609 between the cDNA sequence G124 and A125. For sh-circ-ZNF609 constructs, the target sequence was 5′-AGTCAAGTCTGAAAAGCAATG-3′. All constructs were verified by Sanger sequencing. For circRNA overexpression vectors, the overexpression efficiency and precise cyclization in the back-splicing junction of circRNA were verified by qRT-PCR, agarose gel, and Sanger sequencing.

### 4.13. RNA Pull Down

Endogenous circ-ZNF609 in AC16 cardiomyocytes was pulled down by 5 *μ*g biotinylated DNA oligo antisense junction probe, and the sense junction probe was designed as the negative control. 100 *μ*L Dynabeads MyOne Streptavidin T1 (Invitrogen) was added to each binding reaction and washed 5 times. The packed beads were resuspended into 130 *μ*L phosphate-buffered saline and analyzed by SDS-PAGE and silver staining for differential proteins. Probe sequences used in this study are listed in Table S3.

### 4.14. RNA Immunoprecipitation (RIP)

AC16 cardiomyocytes grown in 10 cm dishes were rinsed twice with pre-ice-cold PBS and harvested. Cell pellets were resuspended in lysis buffer and flash-frozen with liquid nitrogen. Cell lysates were thawed on ice and subjected to 5 rounds of sonication to lyse the cell nucleus. Cell lysate was subjected to centrifugation at 12000g for 30 min at 4°C and precleared by binding to Invitrogen™ Dynabeads Protein G. 1% cell lysate was saved as input. 3 *μ*g anti-rabbit-IgG and YTHDF3 rabbit polyclonal antibody (Proteintech, #25537-1-AP) were incubated with precleared cell lysate at 4°C overnight. 60 *μ*L Invitrogen™ Dynabeads Protein G was washed with NET2 buffer supplied with 200 U/mL RiboLock RNase Inhibitor and 2 mM Ribonucleoside Vanadyl Complexes and then mixed with cell lysate and antibody for another 4 hrs. The beads were collected and washed with NET2 buffer for 4 times. Beads were mixed with 1 mL TRIzol and saved as the IP sample. RNA was isolated by the miRNeasy Mini Kit (QIAGEN) and analyzed by qRT-PCR. For RIP in circ-ZNF609 knockdown, anti-rabbit-IgG and YTHDF1 polyclonal antibody (Proteintech, #17479-1-AP) or YTHDF2 polyclonal antibody (Proteintech, #24744-1-AP) were used. The RIP assay was conducted as the same procedure except AC16 cardiomyocytes were pretransfected with si-circ-ZNF609 or NC control.

### 4.15. m^6^A Methylated RNA Immunoprecipitation- (meRIP-) qPCR

MeRIP-qPCR was performed as previously reported [61]. Briefly, total RNA was isolated by the miRNeasy Mini Kit (QIAGEN) with on-column DNase I treatment. Then, mRNA was purified by oligo(dT) polystyrene beads (Sigma) and fragmented (Invitrogen) at 70°C as the manual recommended. Fragmented mRNA was immunoprecipitated with anti-m^6^A antibody (Synaptic Systems) coupled Dynabeads Protein G (Invitrogen). The enrichment of m^6^A was detected by qPCR; the *Yap* mRNA primer is listed in Table S2.

### 4.16. circ-ZNF609 MS2-MCP Imaging

To validate the nucleocytoplasmic localization of circ-ZNF609, the MS2 macrophage coat protein system was used. NRCMs were cultured in a *μ*-slide 8-well chamber (IBIDI GmbH, Germany) and transfected with designed MS2-tagged circ-ZNF609 plasmid, MS2-GFP-NLS plasmid (addgene #61764), or/and p3MS2 plasmid for 72 hrs. Then, NRCMs were labeled with *α*-actinin while the nucleus was labeled with Hoechst as mentioned in the method of immunofluorescence staining. The images were captured by a confocal microscope (Carl Zeiss, Thuringia, Germany).

### 4.17. RNA Fluorescence *In Situ* Hybridization (FISH)

NRCM was cultured in a *μ*-slide 8-well chamber (IBIDI GmbH, Germany) and fixed with freshly prepared 4% paraformaldehyde at room temperature for 15 minutes. Then, NRCM was permeabilized with 0.5% Triton X-100 and 2 mM Ribonucleoside Vanadyl Complexes (Sigma) on ice for 10 min followed by washing twice with 2x SSC for 10 min each time. The probe (10 ng/mL) was denatured in the hybridization buffer at 90°C for 10 minutes and then immediately chilled on ice for 5 min. NRCM was hybridized and incubated with primary antibodies: *α*-actinin antibody (1 : 200, Sigma) in a hybridization oven at 37°C overnight. After hybridization, the chamber was washed in 2× SSC, 50% formamide for 3 × 5 mins at 42°C, 2× SSC for 3 × 5 mins at 42°C, 1× SSC for 3 × 5 mins at 42°C, 4× SSC for 2 × 10 mins at room temperature, and 2× SSC for 1 hr at 65°C; Cy3-labeled streptavidin and secondary antibodies were added to the NRCM for 1 hr at RT. After washing three times with PBS, Hoechst (1 : 1000, Keygen) was used to label the nucleus for 30 min, and images were captured by a confocal microscope (Carl Zeiss, Thuringia, Germany).

### 4.18. Statistical Analysis

The data were presented as the mean ± SD using GraphPad Prism 8 (GraphPad Software LLC). Statistical analyses were performed by SPSS 20.0 software (independent-sample *t*-test and one-way ANOVA) or GraphPad Prism 8 (two-way ANOVA). To compare differences between two groups, an independent-sample *t*-test was used. Two-way ANOVA with the Tukey test or one-way ANOVA was performed to compare multiple groups. For one-way analysis, the Levene test was used to verify the homogeneity of variance, and the Bonferroni test or Dunnett T3 test was performed according to the results. *P* values less than 0.05 were considered to have statistical significance.

## Data Availability

Data relating to this article are available in the article itself or in its supplementary material online.

## Supplementary Materials

Supplementary Materials Supplementary Figure S1: circ-ZNF609 is resistant to actinomycin D treatment and ubiquitously expressed in adult mouse tissues. Supplementary Figure S2: CLUSTALW alignment of circ-ZNF609 across mammalian species. Supplementary Figure S3: verification of rat circ-ZNF609 overexpression. Supplementary Figure S4: western blot analysis demonstrated the Akt and Erk phosphorylation levels in OGD/R-induced NRCM apoptosis model. Supplementary Figure S5: circ-ZNF609 regulates cardiomyocyte survival via modulating Hippo-YAP. Supplementary Figure S6: circ-ZNF609 regulates cardiomyocyte survival through modulating the crosstalk between Hippo-YAP and Akt signaling. Supplementary Figure S7: circ-ZNF609 mutant with deleted ATGs does not affect the regulatory roles of circ-ZNF609 in cardiomyocytes. Table S1: parameters of echocardiography. Table S2: the primer sequences used for quantitative PCR. Table S3: probe sequences used in this study.
