# Supplementary material for: Downregulation of circ-ZNF609 Promotes Heart Repair by Modulating RNA N6-Methyladenosine-Modified Yap Expression
Source: Research (Wash D C). 2022 Apr 7;2022:9825916. doi: 10.34133/2022/9825916 (PMC9012977; doi:10.34133/2022/9825916)
Supplement: Supplementary Materials — Supplementary Figure S1: circ-ZNF609 is resistant to actinomycin D treatment and ubiquitously expressed in adult mouse tissues. Supplementary Figure S2: CLUSTALW alignment of circ-ZNF609 across mammalian species. Supplementary Figure S3: verification of rat circ-ZNF609 overexpression. Supplementary Figure S4: western blot analysis demonstrated the Akt and Erk phosphorylation levels in OGD/R-induced NRCM apoptosis model. Supplementary Figure S5: circ-ZNF609 regulates cardiomyocyte survival via modulating Hippo-YAP. Supplementary Figure S6: circ-ZNF609 regulates cardiomyocyte survival through modulating the crosstalk between Hippo-YAP and Akt signaling. Supplementary Figure S7: circ-ZNF609 mutant with deleted ATGs does not affect the regulatory roles of circ-ZNF609 in cardiomyocytes. Table S1: parameters of echocardiography. Table S2: the primer sequences used for quantitative PCR. Table S3: probe sequences used in this study. [file 9825916.f1.docx]

Supplementary Materials

**Supplementary Figures**

S1: Circ-ZNF609 is resistant to actinomycin D treatment and ubiquitously expressed in adult mouse tissues.

S2: CLUSTALW alignment of circ-ZNF609 across mammalian species.

S3: Verification of rat circ-ZNF609 overexpression.

S4: Western blot analysis demonstrated the Akt and Erk phosphorylation levels in OGD/R-induced NRCMs apoptosis model.

S5: Circ-ZNF609 regulates cardiomyocyte survival via modulating Hippo-YAP.

S6: Circ-ZNF609 regulates cardiomyocyte survival through modulating the cross-talk between Hippo-YAP and Akt signaling.

S7: Circ-ZNF609 mutant with deleted ATGs does not affect the regulatory roles of circ-ZNF609 on cardiomyocytes.

**Supplementary Tables**

Table S1: Parameters of echocardiography.

Table S2: The primer sequences used for quantitative PCR.

Table S3: Probe sequences used in this study.

**Supplementary Figures:**

**
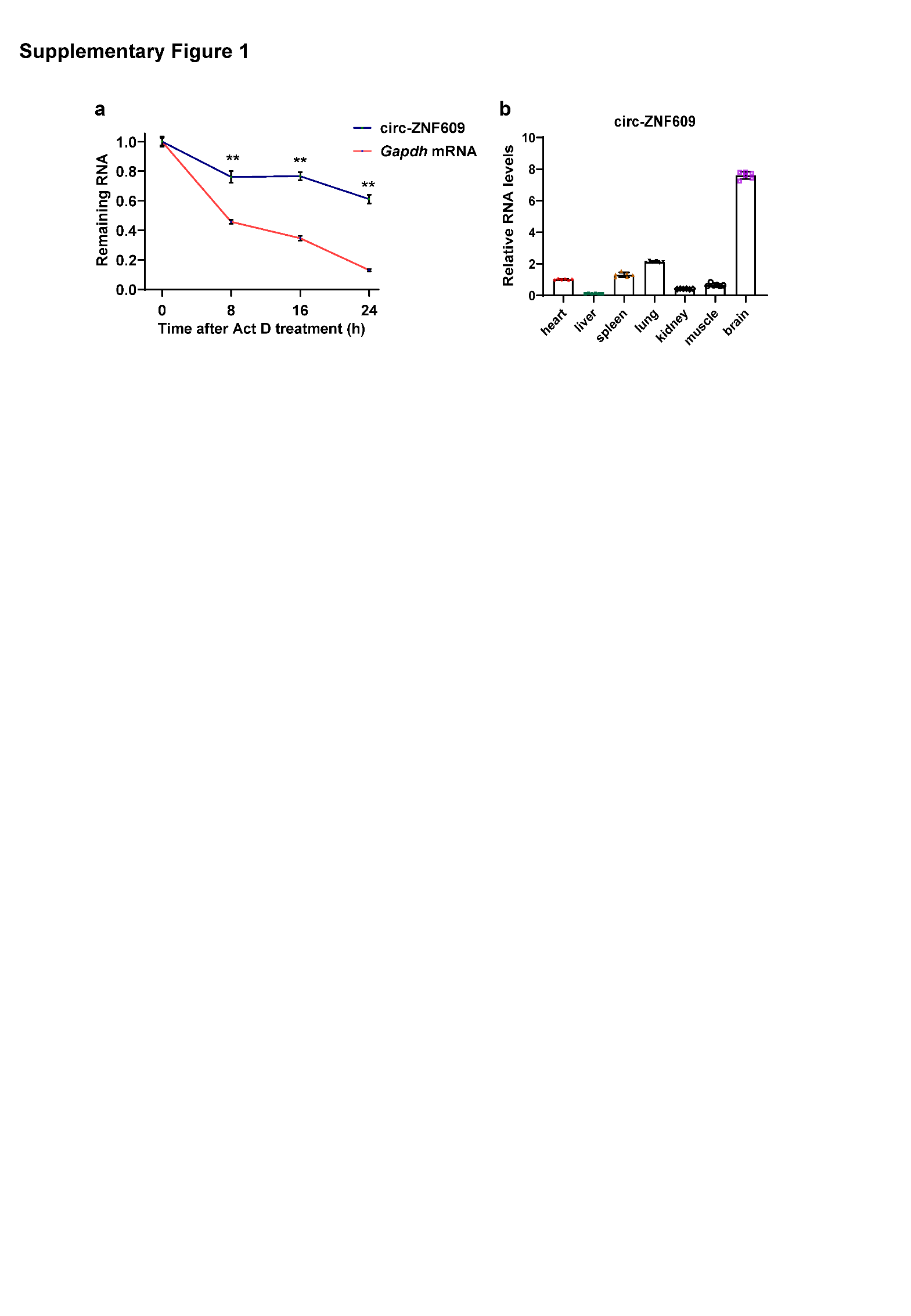
**

**Supplementary Fig. S1. Circ-ZNF609 is resistant to actinomycin D treatment and ubiquitously expressed in adult mouse tissues. (a).** RNA abundance of *Gapdh* mRNA and circ-ZNF609 in AC16 cardiomyocytes were detected after treated with actinomycin D for 0, 8, 16 and 24 hours (**, *P*<0.01, n=6 wells/group). **(b).** The abundance of circ-ZNF609 in adult mouse tissues (n=6/group). Act D, actinomycin D. Data are presented as means±S.D.. (**a,** Independent-sample t test).

**
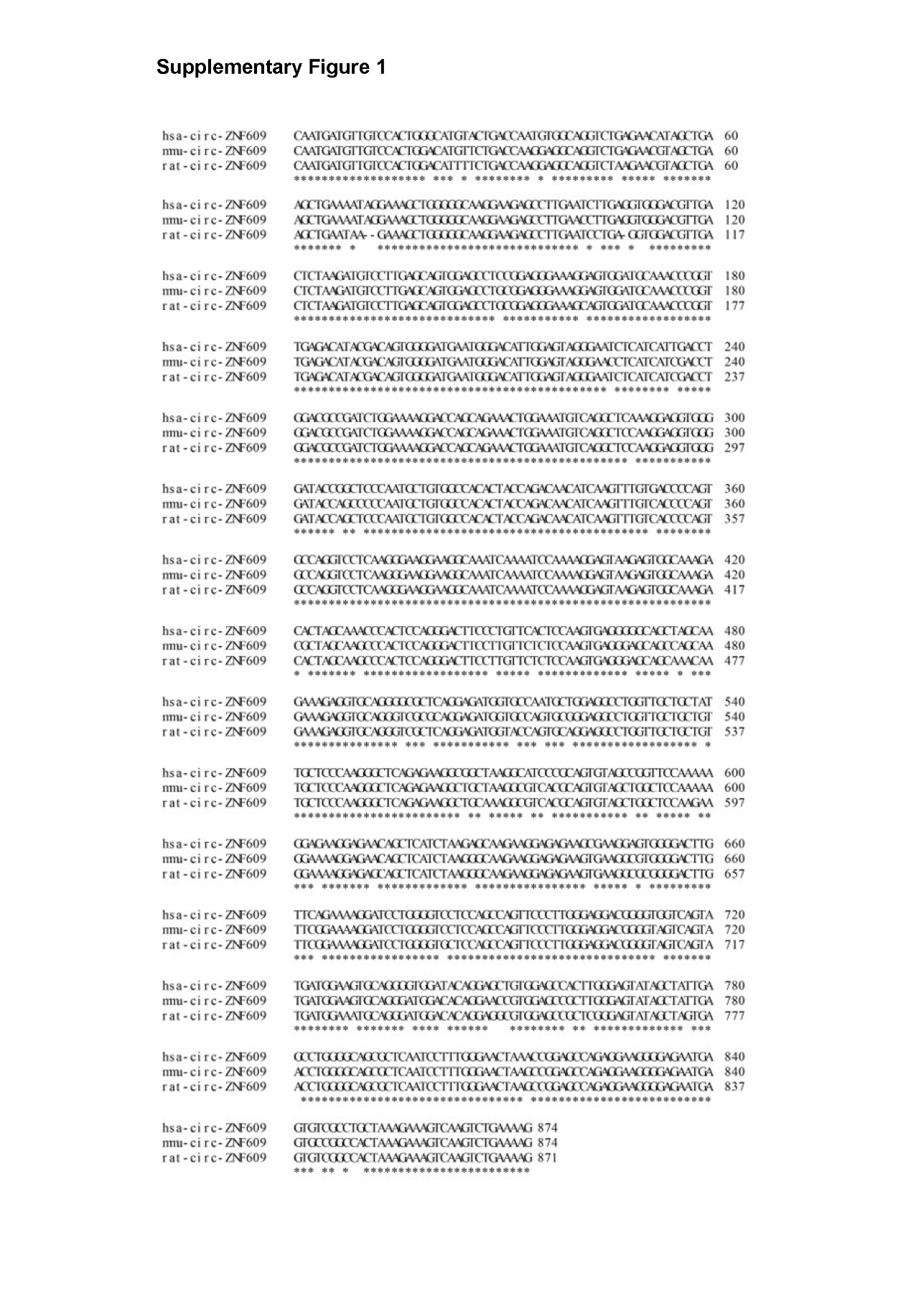
**

**Supplementary Fig. S2.** CLUSTALW alignment of circ-ZNF609 across mammalian species (human, mouse, and rat).

**
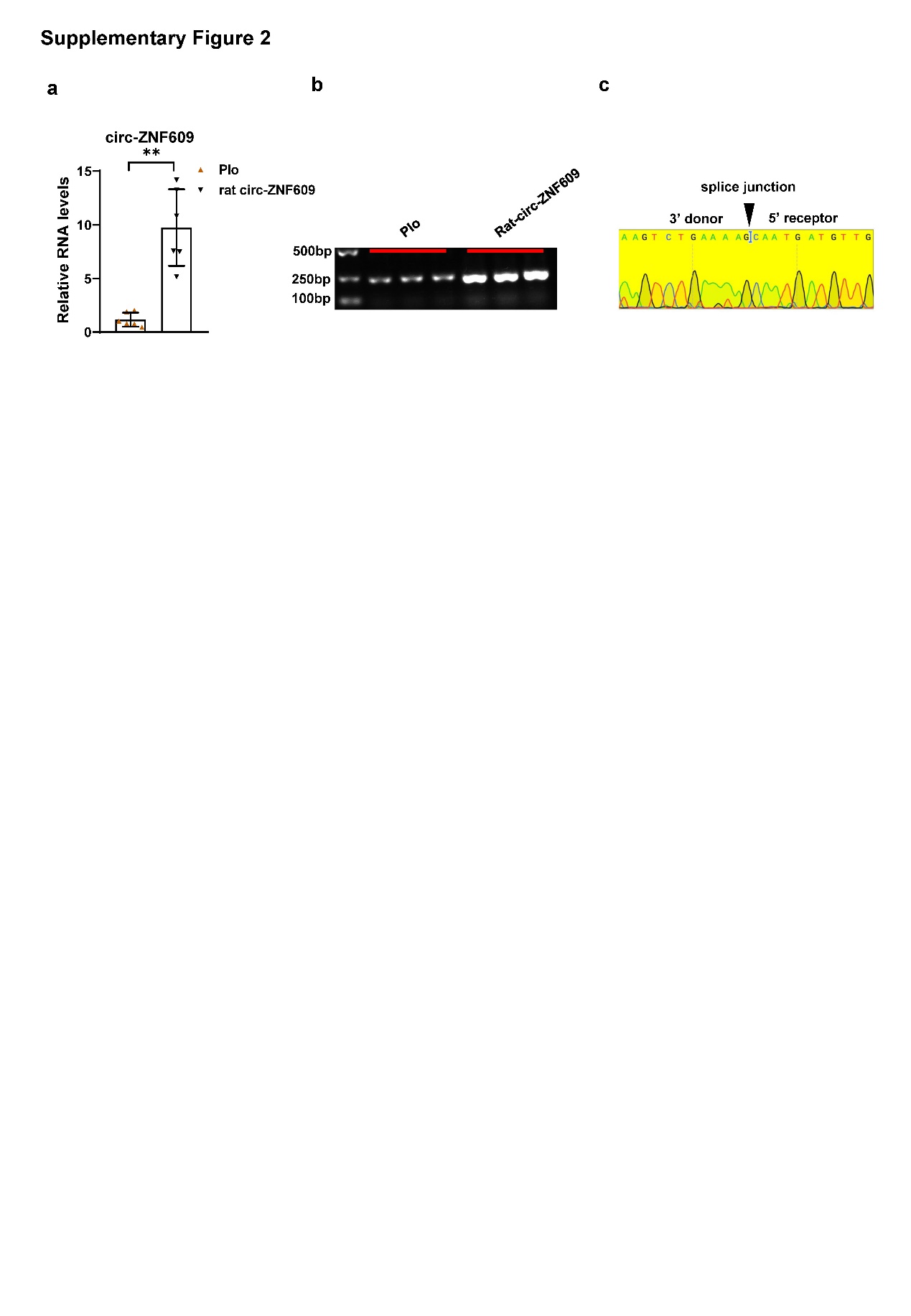
**

**Supplementary Fig. S3.** Verification of rat circ-ZNF609 overexpression by using **(a)** qRT-PCR (**, *P*<0.01, n=6 wells/group), **(b)** agarose gel, and **(c)** sanger sequencing. Data are presented as means±S.D.. (Independent-sample t test).

**
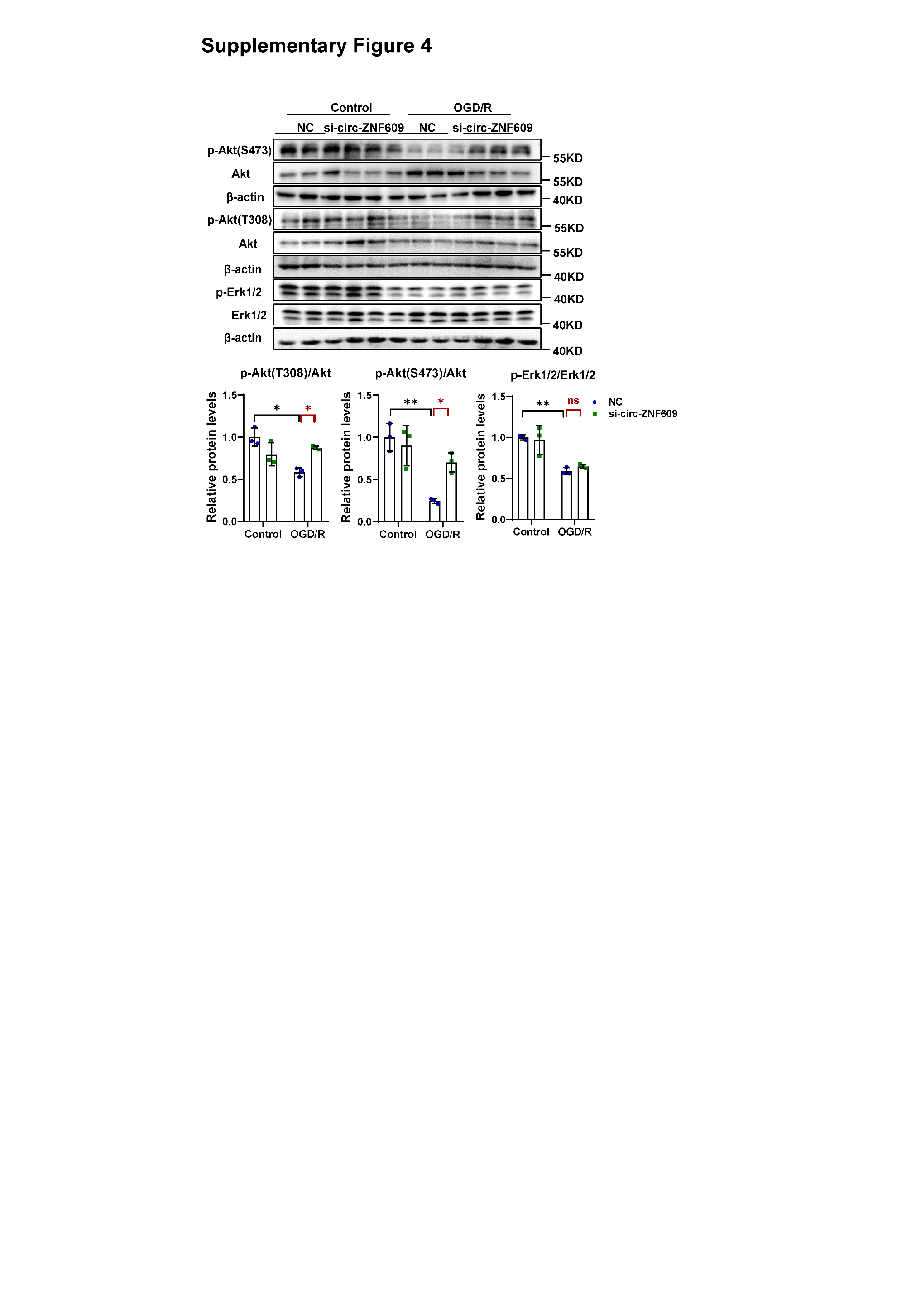
**

**Supplementary Fig. S4.** Western blot analysis demonstrated the Akt and Erk phosphorylation levels in OGD/R-induced NRCMs apoptosis model treated with or without circ-ZNF609 knockdown (**P*<0.05, ***P*<0.01. ns, non-statistically significant. n=3 wells/group). NRCM, neonatal rat cardiomyocyte. OGD/R, oxygen glucose deprivation/reperfusion. NC, siRNA negative control. si-circ-ZNF609, siRNA targeted to circ-ZNF609. Data are presented as means±S.D.. (Two-way ANOVA test).

**
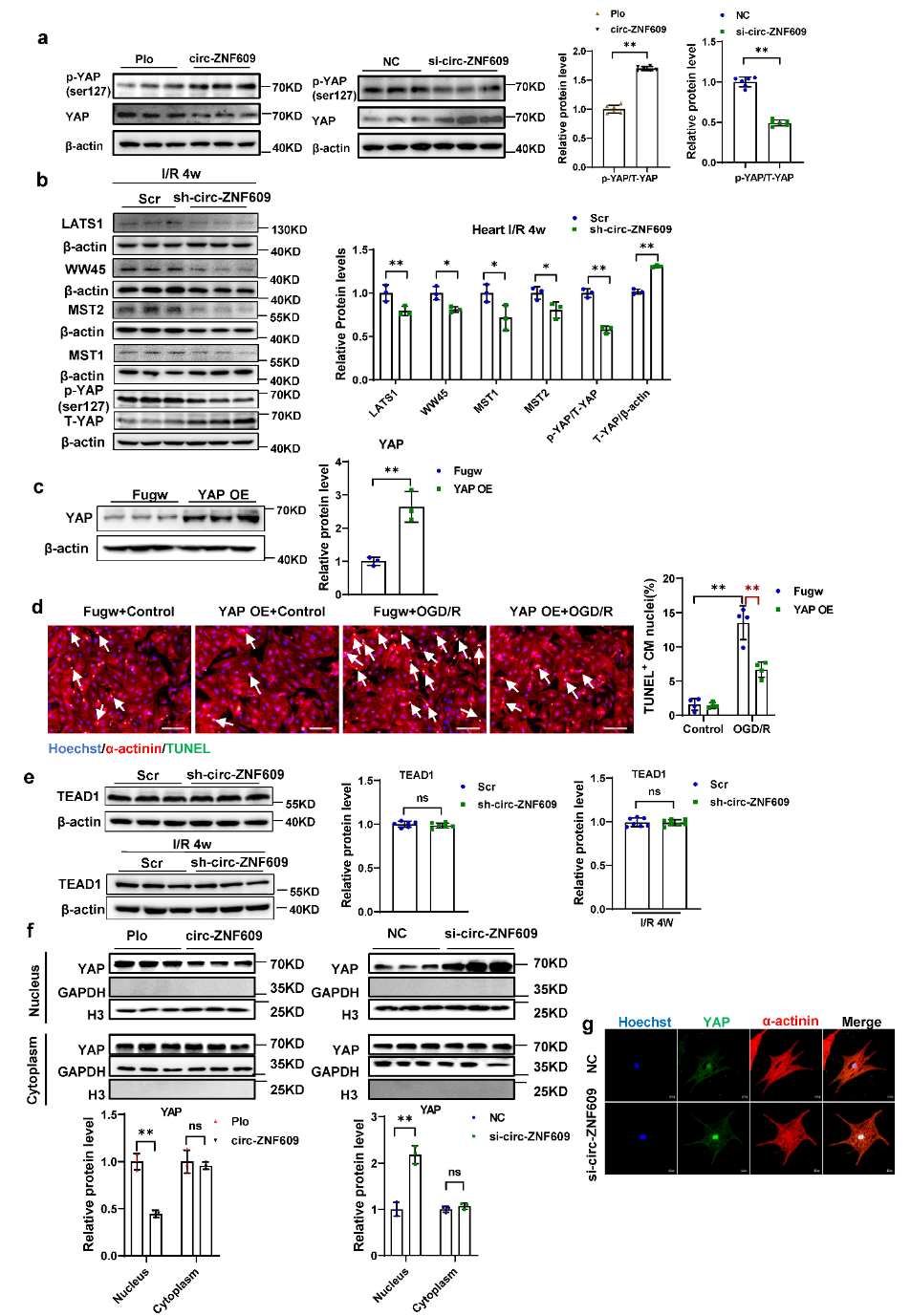
**

**Supplementary Fig. S5. Circ-ZNF609 regulates cardiomyocyte survival via modulating Hippo-YAP. (a)** Western blot analysis of YAP phosphorylation level (S127) in cardiomyocytes after circ-ZNF609 knockdown (si-circ-ZNF609) or circ-ZNF609 overexpression (circ-ZNF609) (***P*<0.01, n=6 wells/group). **(b)**. Western blot analysis showed the decreased expression of Hippo components LATS1, WW45, MST1/2, Total YAP(T-YAP) and YAP phosphorylation level in mice I/R remodeling heart after treated with AAV9-sh-circ-ZNF609 (**P*<0.05, ***P*<0.01, n=3 mice/group). **(c)**. Verification of rat YAP overexpression by Western blot (***P*<0.01, n=3 wells/group). **(d)**. Representative images of immunofluorescence staining and quantification of the relative TUNEL positive NRCMs showed that YAP overexpression resist to NRCMs apoptosis (***P*<0.01, n=4 wells/group, scale bar=100μm). **(e)** Western blot analysis the expression of TEAD1 in mice sham or I/R remodeling heart after treated with AAV9-sh-circ-ZNF609 (ns, non-statistically significant, n=6 mice/group). **(f)** Western blot analysis the distribution of YAP in nucleus and cytoplasm in NRCM after circ-ZNF609 knockdown (si-circ-ZNF609) or circ-ZNF609 overexpression (circ-ZNF609) (***P*<0.01, ns, non-statistically significant. n=6 wells/group). **(g)** Coimmunofluorescence staining of YAP (Green), α-actinin (Red) and Hoechst (Blue) in NRCMs with or without circ-ZNF609 knockdown (scale bar=10μm). OGD/R, oxygen glucose deprivation/reperfusion. NC, siRNA negative control. si-circ-ZNF609, siRNA targeted to circ-ZNF609. Scr, shRNA scramble control. sh-circ-ZNF609, shRNA targeted to circ-ZNF609. Plo, circRNA overexpression empty vector Plo-ciR without insert circ-ZNF609 sequence. circ-ZNF609, circ-ZNF609 overexpression construct. Fugw, empty vector without YAP overexpression. YAP OE, YAP overexpression construct. Data are presented as means±S.D. (**a**, **b**, **c, e, and f,** independent-sample t test. **d,** Two-way ANOVA test.).

**
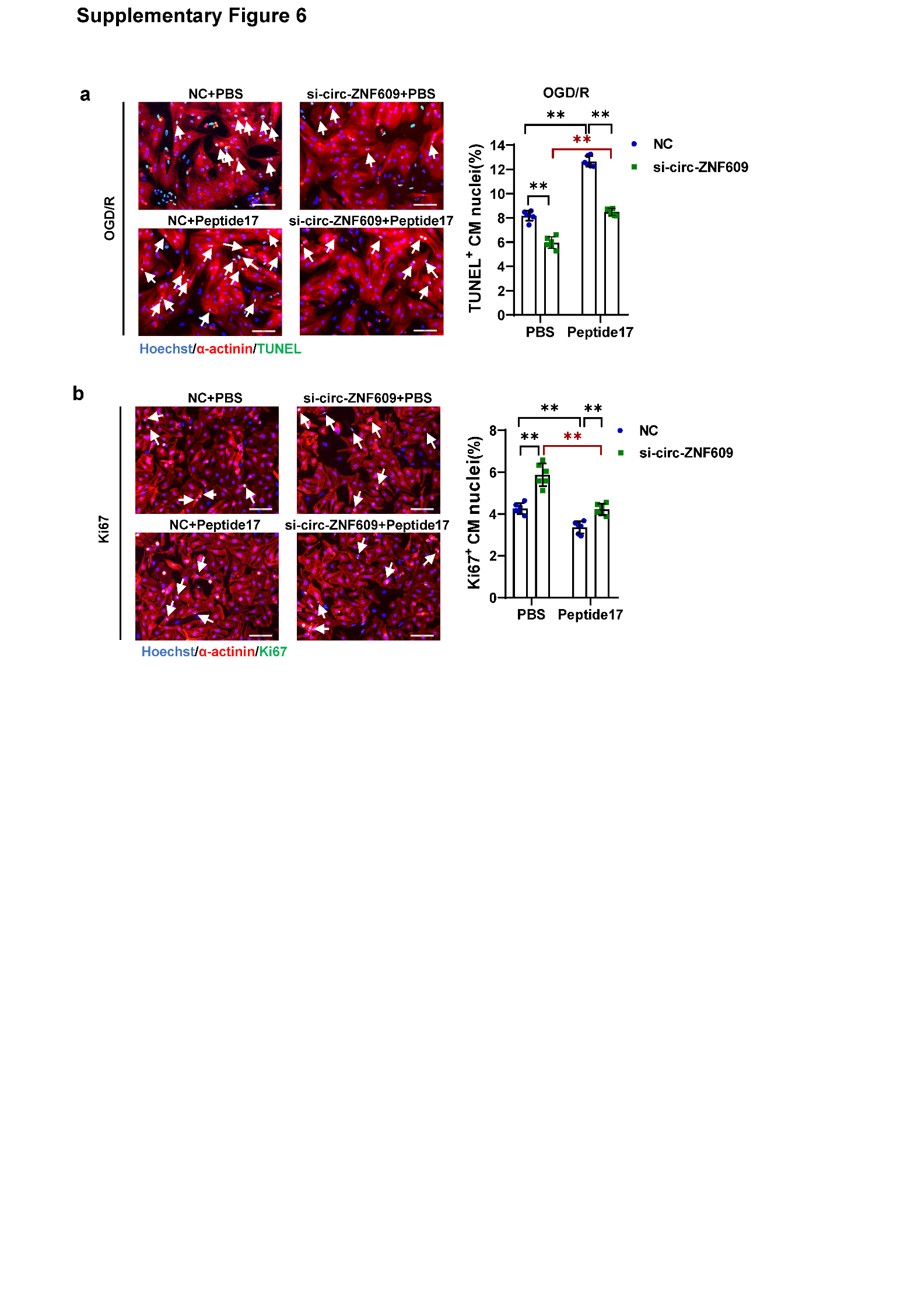
**

**Supplementary Fig. S6. Circ-ZNF609 regulates cardiomyocyte survival through modulating the cross-talk between Hippo-YAP and Akt signaling.** Representative images of immunofluorescence staining and quantification of the relative TUNEL positive **(a)** and Ki67 positive **(b)** NRCMs showed YAP/TEAD inhibitors Peptide17 could both blunt the anti-apoptosis and pro-proliferation effects of circ-ZNF609 knockdown in NRCM. (***P*<0.01, n=6 wells/group, scale bar=100μm). OGD/R, oxygen glucose deprivation/reperfusion. TUNEL, terminal deoxynucleotidyl transferase dUTP nick end labeling. Data are presented as means±S.D. (**a**, and **b**, Two-way ANOVA test.).

**
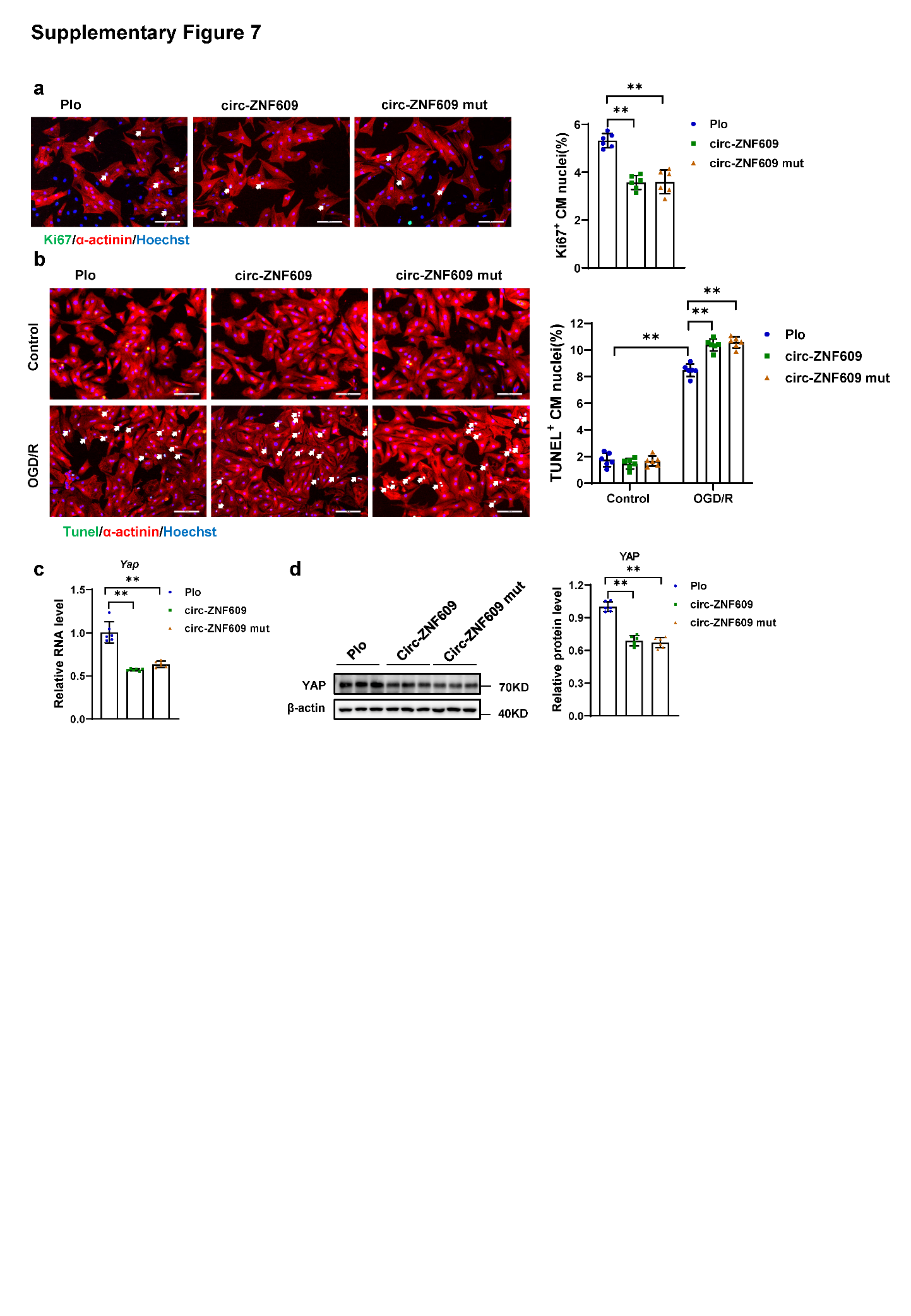
**

**Supplementary Fig. S7. Circ-ZNF609 mutant with deleted ATGs does not affect the regulatory roles of circ-ZNF609 on cardiomyocytes. (a-b)** Representative images of immunofluorescence staining and quantification of the relative Ki67 positive **(a)** and TUNEL positive **(b)** NRCMs showed that circ-ZNF609 mutant (rat-circ-ZNF609Δ1-2) construct preserved the regulation of circ-ZNF609 on proliferation and OGD/R-induced apoptosis in NRCMs. (***P*<0.01, n=6 wells/group, scale bar=100μm). **(c-d)** qRT-PCR and western blot analysis revealed that circ-ZNF609 mutant (hs-circ-ZNF609Δ1-2) construct preserved the regulation of circ-ZNF609 on YAP in AC16 cardiomyocytes. (***P*<0.01, n=6 wells/group). OGD/R, oxygen glucose deprivation/reperfusion. TUNEL, terminal deoxynucleotidyl transferase dUTP nick end labeling. Plo, circRNA overexpression empty vector Plo-ciR without insert circ-ZNF609 sequence. circ-ZNF609, circ-ZNF609 overexpression construct. Data are presented as means±S.D. (**a**, **b**, and **d**, one-way ANOVA followed by Bonferroni test. **c**, one-way ANOVA followed by Dunnett T3 test.).

**Supplementary Tables:**

**Table S1**

Parameters of echocardiography.

|  | Sham | | I/R 4w | |
| --- | --- | --- | --- | --- |
|  | AAV9-Scramble | AAV9-sh-circ-ZNF609 | AAV9-Scramble | AAV9-sh-circ-ZNF609 |
| Heart rate (BPM) | 461.05±16.62 | 433.38±23.87 | 453.75±46.07 | 451.51±37.81 |
| LVESD (mm) | 2.53±0.27 | 2.61±0.94 | 2.86±0.26^**^ | 2.84±0.35 |
| LVEDD (mm) | 3.73±0.13 | 3.71±0.39 | 3.69±0.40 | 4.01±0.27 |
| LV Volume; s (μl) | 23.39±5.97 | 25.75±9.03 | 31.55±6.94^**^ | 31.46±10.25 |
| LV Volume; d (μl) | 59.60±4.80 | 59.50±14.05 | 58.92±13.35 | 70.97±11.49 |
| LVAW; s (mm) | 1.16±0.18 | 1.20±0.14 | 1.03±0.10 | 1.17±0.20 |
| LVAW; d (mm) | 0.74±0.08 | 0.84±0.13 | 0.85±0.18 | 0.82±0.10 |
| LVPW; s (mm) | 1.16±0.20 | 1.05±0.18 | 0.98±0.27 | 1.11±0.14 |
| LVPW; d (mm) | 0.84±0.15 | 0.81±0.15 | 0.75±0.25 | 0.79±0.14 |
| LV Mass (mg) | 103.93±20.0 | 107.61±13.45 | 102.48±13.09 | 118.84±17.17 |
| LV Mass Cor (mg) | 83.15±16.0 | 86.09±10.76 | 81.98±10.47 | 95.07±13.73 |
| Stroke Volume (μl) | 36.21±4.30 | 33.75±5.77 | 24.51±7.76^**^ | 39.51±4.87^##^ |
| Cardiac output (ml/min) | 16.72±2.30 | 14.56±2.31 | 10.27±3.17^**^ | 17.91±3.08^##^ |

Abbreviation: BPM, beat per minute; LVESD, left ventricular end-systolic dimension; LVEDD, left ventricular end-diastolic dimension; LV Volume s, left ventricular volume systolic; LV Volume d, left ventricular volume diastolic; LVAWs, left ventricular anterior wall thickness systolic; LVAWd, left ventricular anterior wall thickness diastolic; LVPWs, left ventricular posterior wall thickness systolic; LVPWd, left ventricular posterior wall thickness diastolic; LV Mass, left ventricular mass index.

Data are presented as mean±SD. ^**^, P<0.01 compared to AAV9-Scramble+Sham; ^##^,P<0.01 compared to AAV9-Scramble+I/R 4W. (Two-way ANOVA, Tukey)

**Table S2**

The primer sequences used for quantitative PCR were as follows:

| gene | Forward | Reverse | |
| --- | --- | --- | --- |
| m-*Gapdh* | AGGTCGGTGTGAACGGATTTG | TGTAGACCATGTAGTTGAGGTCA | |
| m-*18s* | TCAAGAACGAAAGTCGGAGG | GGACATCTAAGGGCATCAC | |
| m-*α-SMA* | CCCAGACATCAGGGAGTAATGG | TCTATCGGATACTTCAGCGTCA | |
| m-*Col1a1* | GCTCCTCTTAGGGGCCACT | CCACGTCTCACCATTGGGG | |
| m-*Col3a1* | CTGTAACATGGAAACTGGGGAAA | CCATAGCTGAACTGAAAACCACC | |
| m-*Ctgf* | CTTCTGCAGACTGGAGAAGC | CAGCCAGAAAGCTCAAACTTG | |
| rm-circ-ZNF609 | CCTTTGGGAACTAAGCCGGA | GTCTCAACCGGGTTTGCATC | |
| m-circ-ZNF609 | GAAGGGGAGAATGAGTGCCG | GTCAACGTCCCACCTCAAGGTTC | |
| m-*ZNF609*-linear#1 | GAAGGGGAGAATGAGTGCCG | TGACCACTGGCACTAACAGG | |
| m-*ZNF609*-linear#2 | CCCTAACTCCCCTCCACGTA | TCCTTCGACTTGGCACTGTC | |
| m-*Anp* | TCTTCCTCGTCTTGGCCTTT | CCAGGTGGTCTAGCAGGTTC | |
| m-*Bnp* | TGGGAGGTCACTCCTATCCT | GGCCATTTCCTCCGACTTT | |
| rat-*18s* | ATTCGAACGTCTGCCCTATCAA | CGGGAGTGGGTAATTTGCG |  |
| hs-circ-ZNF609 | AAACCGGAGCCAGAGGAAGG | CAGCTATGTTCTCAGACCTGC |  |
| hs-*Yap* | TCACAGCAGAACCGTTTCCC | CCAGTGTTCCAAGGTCCACA |  |
| hs-*Ythdf1* | CAAGCACACAACCTCCATCTTCG | GTAAGAAACTGGTTCGCCCTCAT |  |
| hs-*Ythdf2* | TAGCCAGCTACAAGCACACCAC | CAACCGTTGCTGCAGTCTGTGT |  |
| hs-*Ythdf3* | TCAGAGTAACAGCTATCCACCA | GGTTGTCAGATATGGCATAGGCT |  |

**Table S3**

Probe sequences used in this study.

| Probe | Sequences |
| --- | --- |
| circ-ZNF609 Antisense probe | 5’-Biotin-aaaTGGACAACATCATTGCTTTTCAGACTTGAC |
| circ-ZNF609 Sense probe | 5’-Biotin-aaaGTCAAGTCTGAAAAGCAATGATGTTGTCCA |
